# Reasons for shisha smoking: Findings from a mixed methods study among adult shisha smokers in Nigeria

**DOI:** 10.1371/journal.pgph.0002853

**Published:** 2024-02-02

**Authors:** Noreen Dadirai Mdege, Ranti Ekpo, Sharon Ogolla, Seember Joy Ali, Aminata Camara, Esther Mugweni

**Affiliations:** 1 Department of Health Sciences, University of York, York, United Kingdom; 2 Development Gateway: An IREX Venture, Washington, DC, United States of America; 3 Centre for Research in Health and Development, York, United Kingdom; 4 Voice of Children Foundation, Abuja, Nigeria; 5 Centre for Health Services Studies, University of Kent Canterbury, Kent, United Kingdom; University of the Philippines Diliman, PHILIPPINES

## Abstract

Shisha smoking has increased significantly worldwide over the past decade including in developing countries such as Nigeria. We aimed to understand the reasons for shisha smoking in Nigeria in order to address the lack of context-specific evidence to inform the national response to the growing threat posed by shisha smoking. We adopted the Theory of Planned Behaviour to conduct in-depth interviews among 78 purposely sampled current shisha smokers in 13 states (six in each state), and a quantitative survey including a random sample of 611 current shisha smokers in 12 states, across the six geopolitical zones in Nigeria. The in-depth interview data was analysed using thematic analysis whilst the quantitative survey data was analysed descriptively. We triangulated the key findings from the two datasets using a triangulation matrix organised by the three meta-themes: attitude, subjective norms, perceived behavioural control. Positive attitudes towards shisha smoking stem from shisha flavours, perceived pleasure from shisha smoking, curiosity about product attributes, beliefs about health benefits, limited knowledge on the health effects, and weak regulation. Having friends and family members who smoke shisha and the need to belong, particularly during social events, also promote shisha smoking. Negative societal views towards shisha smoking are potentially a protective factor. The availability of and ability to smoke shisha in many places makes shisha more accessible, whilst the high costs of shisha are potentially prohibitive. The findings also indicate that quitting shisha smoking without support is difficult. Restrictions on flavours, strengthening compliance monitoring and enforcement of the tobacco control laws in relation to shisha (e.g., smoke-free environments in indoor and outdoor public places; health warnings in English on shisha products including the pots; and tax and price measures) have the potential to minimise initiation and use, and to protect the health and wellbeing of Nigeria’s general public.

## Introduction

Shisha smoking is a form of tobacco smoking where the tobacco, flavoured or unflavoured, is combusted using charcoal or briquettes and the tobacco smoke is inhaled after it has passed through water or other liquids [1]. Although currently not as common as cigarette smoking, the prevalence of shisha smoking is increasing worldwide including in developing countries, especially among young people [2–7]. In Nigeria, data from the 2018 Nigeria Demographic and Health Survey (NDHS) suggests that 0.2% of Nigerians aged 15 to 59 years old smoke shisha [8]. However, studies among secondary school pupils and university students have reported high shisha smoking prevalence rates ranging from 3% to 7% [5,9,10].

Shisha smoking poses significant health hazards including a number of cancers, reduced lung function, and cardiovascular problems [11,12]. Shisha smoking also results in adverse pregnancy outcomes including respiratory diseases and low birth weight in babies born to mothers who smoke shisha [11,12]. This means that the high prevalence of shisha smoking among youths in Nigeria has significant health, social, and economic consequences. The World Health Organization Framework Convention on Tobacco Control (WHO FCTC) supports nations to address the tobacco epidemic and its consequences by providing various evidence-based policy interventions such as tobacco taxes, smoke-free public places and bans on tobacco advertising, promotion and sponsorship [13]. Nigeria’s Federal Government signed the convention agreement in 2005, and in 2015 signed into law the National Tobacco Control (NTC) Act which regulates all aspects of tobacco control in Nigeria [4–6,14]. Nigeria’s National Tobacco Control Regulations, 2019, provide a legal framework for the effective implementation, and achievement of the objectives of the Act [15]. Nigeria has embraced the MPOWER initiative which supports member states to implement demand reduction measures and report progress toward targets.

Strengthening the national response to the growing threat posed by shisha smoking needs to be informed by context-specific data. Unfortunately, the studies that have investigated shisha smoking in Nigeria are of limited scope because they focus on subgroups of the population such as secondary school and university students, medical professionals or nightclub patrons [5,6,9,10,16–20]. Most of them also have a small sample size, or only cover a few geographical areas, which limits their internal and external validity. In addition, most of these studies are quantitative cross-sectional studies with only two qualitative studies identified: one with 20 participants in total across three focus group discussions in Lagos [19], and the other with two participants in in-depth interviews and 12 participants in total across two focus group discussions at the University of Ibadan [9]. This limits our understanding of the context within which shisha smoking occurs, experiences of shisha smokers, and other important factors that influence shisha smoking or cessation. Understanding these issues is important when developing effective policies or interventions to tackle shisha smoking.

To address these evidence gaps, we used mixed methods research to investigate the factors associated with shisha smoking, and shisha use patterns, attitudes and behaviours among general- population adults (aged 18 years and above) in 13 states across all six geopolitical zones in Nigeria. Here we report the reasons for smoking shisha that were cited by current shisha smokers in our study sample. Other study results, including statistical analysis to identify factors associated with shisha smoking, will be reported separately. The information generated from this study can support the development of policy- or individual-level strategies to reduce shisha smoking in Nigeria and other similar contexts.

## Methods

### Overall study design

This was a sequential, mixed-methods cross-sectional study where in-depth qualitative interviews were conducted between 18^th^ December 2021 and 21^st^ January 2022, followed by a quantitative survey between 28^th^ July and 11^th^ September 2022. For this study, mixed-methods were used for two reasons: 1) in order for utilise findings from the qualitative component to inform the questions that were important to include in the questionnaire for the quantitative component; and 2) to cross-validate data from the two sources and increase confidence in the study findings [21]. We adopted the Theory of Planned Behaviour (TPB) which has been used widely to explore tobacco use, including shisha smoking [22–25], and assumed that intention (participants’ readiness to smoke shisha) preceded shisha smoking (behaviour). The intention to smoke shisha was determined by attitude (positive or negative evaluations of smoking shisha), subjective norms (the perceived social pressure/support to smoke or quit shisha), and perceived behavioural control (the participant’s evaluations of the ease or difficulty of smoking or stopping smoking shisha) [26,27]. Our data collection methods and analysis explored the determinants of intentions to smoke shisha to clearly articulate areas that could be targeted by future policies or interventions to reduce shisha smoking in Nigeria.

### Ethics approval and informed consent

The qualitative component of the study was approved on 15 December 2021 (Approval Number NHREC/01/01/2007-15/12/2021), whilst the quantitative component was approved on 21 July 2022 (Approval Number NHREC/01/01/2007-21/07/2022) by the National Health Research Ethics Committee, Nigeria (NHREC). Each research participant gave written consent before participating in the study, after having read and understood the study information.

### Study population and sample size

Participants had to be 18 years and older. In addition, to be eligible for the qualitative interviews, a participant had to be a self-reported current shisha smoker who had lived in the study area for the past 6 months. We aimed for 78 shisha smokers, 6 in each participating state. This number allowed us to reach data saturation in terms of scope and replicability [28]. For the quantitative component, participants were eligible if they lived in the study area and their phone number was listed in the 2018/19 Nigeria Living Standard Surveys (NLSS) sample frame. Using simulations and rule of events per variable approaches, we needed a minimum of 1200 participants of which 600 would be current shisha smokers. These approaches have been shown to produce accurate and powered estimates derived from estimated samples that represent the target population parameters [29–31]. We assumed a 15% response rate which is consistent with the phone survey experience of other studies in Nigeria [32]. We therefore needed to call about 8,000 phone numbers in our sample frame to achieve the desired survey sample size.

### Sampling procedures

**Fig 1:**
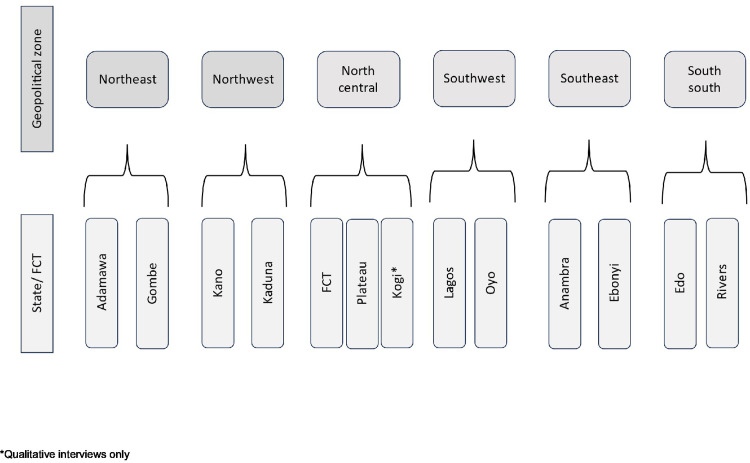
Study sites.

Using the urbanisation rate of each state as a proxy for the prevalence of shisha smoking [6,33], we selected the two most urbanised states (including the Federal Capital Territories (FCT)) in each of the six geopolitical regions using data from the 2018 Nigeria Demographic and Health Survey (NDHS) [8]. In cases where the most urbanised state was judged as difficult to access, for example, due to recurring insecurity challenges (e.g., Borno state), it was replaced by the state with the next highest urbanisation rate. For the qualitative interviews, we had one additional state. Therefore 13 states were included in the qualitative interviews, whilst 12 states were included for the quantitative survey (Figs 1 and S1).

For the qualitative interviews, at least one urban and one rural/semi-urban location were chosen in each state. We then used purposive sampling [34] to enrol current shisha smokers from public places that are well known for shisha smoking such as social clubs, nightclubs, bars, pubs, cafes, and restaurants. Those who were enrolled in the study were asked to refer any current shisha smokers that they know to the study team.

For the quantitative survey, our sampling frame was the 2018/19 NLSS, which contains a list of households that is representative at national, zonal, and state levels including FCT and contains phone numbers of up to 3 household members. Two-stage stratified sampling was adopted within each participating state. First, NLSS enumeration Areas (EAs) were stratified into urban or rural, and seven and three EAs randomly selected from the urban and rural stratum, respectively. In the second stage, in every participating EA, phone numbers were randomly selected and called until 10 individuals (5 current shisha smokers and 5 non-smokers) had participated in the survey. This was complemented by snowball sampling to enrol adequate numbers of current shisha smokers- the recruitment rate for this group was too low to achieve the required numbers using random sampling. About 3 out of every 10 individuals sampled were females.

### Data collection

Data collection tools were developed based on a review of available literature and the TPB. The quantitative survey questionnaire was also additionally informed by the findings from the qualitative interviews: some of the questions included in the questionnaire were derived from the themes/subthemes identified in the qualitative data (see S1 Text). Data collection was conducted in English, as well as four major local languages, i.e., Hausa, Igbo, Yoruba, and Pidgin English. English data collection tools were translated to these languages and pilot tested before use.

#### Qualitative interviews

In each state, the interviews were conducted by two experienced qualitative researchers who were fluent in the local language. The field team received a 2-day training on the study protocol, data collection tools, and research ethics. Interviews were conducted using an interview guide that included the following topics: history of shisha smoking, shisha smoking histories of family members and friends, perceived benefits from smoking shisha, awareness of health implications of smoking shisha, perceptions of health risks of shisha smoking, use of other psychoactive substances, and regulations on shisha smoking in Nigeria (S2 Text). Each interview took between 40 and 60 minutes. As reimbursement for their time, each of the facility managers at the participant recruitment locations was provided with 2,000 Naira (∼4.8 USD) and each study participant received 500 Naira (∼1.2 USD). These amounts are aligned with research participant reimbursement expectations in Nigeria, and were approved by NHREC.

#### Quantitative survey

The questionnaire included current shisha smoking status which was determined from two questions: 1) *“Have you ever smoked shisha*, *even one or two puffs*?*”*, plus 2) *“Do you currently smoke shisha on a daily basis*, *less than daily or not at all*?*”* (S1 Text). Those who responded “no” to the first question were classified as shisha never smokers. Those who responded “yes” to the first but “not at all” to the second question were classified as past smokers of shisha, whilst those who responded “daily” or “less than daily” to the second question were classified as current shisha smokers. Never smokers and past smokers of shisha made up the current shisha nonsmokers. Demographic and socioeconomic questions included age, gender, rural/urban residence, level of education, religion, employment status, and asset-based wealth based on household ownership of 12 assets [8,35]. Other variables obtained from literature and/or the qualitative interviews included questions relating to social perceptions about shisha smoking such as the number of family members and closest friends who smoke shisha, as well as questions on cigarette smoking and alcohol consumption in the past year [31,35–39]. Perceived stress was measured using the 4-item Perceived Stress Scale (PSS-4) [40–42], anxiety using the General Anxiety Disorders-2 (GAD-2) [43], and depression using the Patient Health Questionnaire-2 (PHQ-2) [44].

For current shisha smokers, we also measured shisha use patterns, including frequency and quantity smoked, smoking locations, and concurrent smoking of shisha with other psychoactive substances [8,35,45]. Attitudes towards shisha smoking among current shisha smokers were measured using adapted questions from the Attitudes Toward Smoking Scale (ATS-18) [46]. Additional questions on knowledge and health perceptions of shisha were identified from literature [36–38]. We used the self-efficacy questionnaire (SEQ-12) to assess the ability of current shisha smokers to resist smoking shisha in specific situations (therefore perceived behavioural control) [47]. In addition, there were selected GATS questions on shisha smoking cessation including quit attempt and quit intentions [35]. The influence of the media on shisha smoking and knowledge of regulations on shisha in Nigeria were also assessed.

The questionnaires were programmed using the SurveyCTO software. Data was collected from a call centre using Computer Assisted Telephone Interviews (CATI) by 23 data collectors who had received a 5-day training including technical content on the questionnaire and its administration, and research ethics. The training involved classroom training, role-playing, as well as field testing. The questionnaire took up to 35 minutes to administer. Each participant received five hundred Naira (∼1.2 USD) worth of airtime sent directly to their mobile phone as compensation for their time.

### Data analysis

#### Qualitative interviews

Interviews were transcribed verbatim and non-English transcripts translated into English. ATLAS-ti software was used for data management. All interview data were analysed using Thematic Analysis guided by the TPB framework. We familiarised ourselves with the data, generated initial codes, reviewed the codes, defined and named the themes, and revised these themes to develop a matrix that was used for the final analysis [34,48]. Intercoder reliability was secured by the collaborative development of the codebook and ensuring that all the analyses were done using the same codebook and analysis matrix [48].

#### Quantitative survey

Descriptive analysis was conducted to produce frequencies and proportions for categorical variables, and means (SDs) for continuous variables. All analyses were performed using STATA statistical software. We presented the results in tables, graphs and figures using Microsoft Word and Excel.

#### Triangulation of qualitative and quantitative findings

We triangulated the key findings from the two datasets using a triangulation matrix (S1 Table) organised by the three meta-themes [49] according to the TPB: attitude, subjective norms, perceived behavioural control [26,27]. We mapped data and results from each of the two datasets onto the relevant meta-themes. Data in each meta-theme was also arranged into sub-themes identified from the two datasets. For each meta-theme and sub-theme, at least one dataset provided data. Where both datasets provided findings, we compared them to consider if they were convergent (in agreement), complementary (partial agreement), or contradictory (disagreement) [49].

## Findings

### Sample characteristics

#### Qualitative interviews

The characteristics of the 78 respondents in the qualitative interviews are summarised in Table 1.

**Table 1:** Qualitative interview respondents’ socio-demographic characteristics.

Variable	Frequency	Percentage (N = 78)
**Age**		
<30 (Younger Adult)	41	52.6
30+ (Older Adult)	37	47.4
**Gender**		
Male	40	51.3
Female	38	48.7
Level of education*		
High	44	56.4
Low	34	43.6
**Religion**		
Christian	62	79.5
Muslim	10	12.8
Not Known	6	7.7
**Location**		
Rural/Semi-urban	30	38.5
Urban	48	61.5

*Level of education was used as a proxy for respondents’ socio-economic status (SES). *It was categorized as 1) Low: Secondary education and below 2) High: Above secondary education*.

#### Quantitative survey

611 current shisha smokers participated in the quantitative survey. The mean age was ∼29 years (Table 2). 77% were male, 77% lived in urban EAs, 66% were Christian, and 72% were currently employed. For 41%, the highest level of education was secondary/post-secondary. The poorer quintile had the highest numbers of current shisha smokers (196; 32%). North-Central and North-West zones had the highest number of current shisha smokers (each with 126; 21%).

**Table 2:** Quantitative survey respondents’ socio-demographic characteristics.

Variables	Frequency	Percentage (N = 611)
**Age in single years:** mean (SD)	28.8 (6.0)	N/A
**Gender**:		
Female	143	23.4
Male	468	76.6
**Place of residence:**		
Rural	139	22.8
Urban	471	77.2
**Highest level of education:**		
Primary School or Less	11	1.8
Secondary school	390	63.8
Post-secondary school	209	34.2
Refused to answer	1	0.2
**Religion:**		
Christianity	402	65.8
Muslim	198	32.4
Traditional/None	11	1.8
**Wealth Index:**		
Poorest	183	30.0
Poorer	196	32.2
Middle	87	14.3
Richer	89	14.6
Richest	55	8.9
**Work status:**		
Currently unemployed	3	5.3
Currently employed	436	71.5
Student	106	17.4
Apprentice	35	5.8
**Zone:**		
North-Central	126	20.7
North-East	103	16.8
North-West	126	20.7
South-East	77	12.6
South-South	94	15.4
South-West	84	13.8

**Fig 2:**
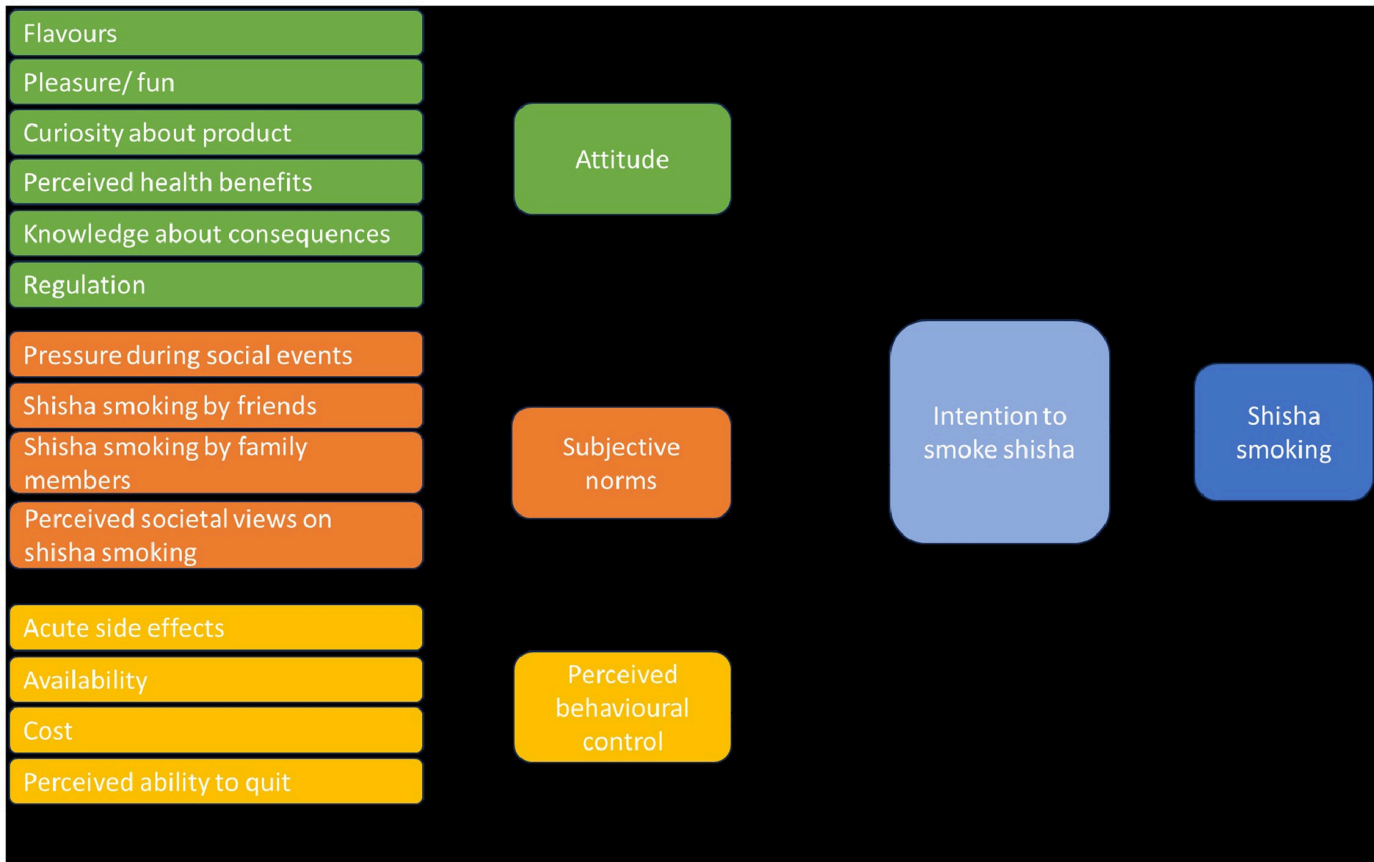
Logic model for reasons for shisha smoking.

None of our qualitative in-depth interview participants expressed a desire to quit shisha smoking in the next month, and only 5% (31/611) of participants in the quantitative survey expressed that they were thinking of quitting in the next month. Almost all participants in the sample were, therefore, intending to continue to smoke shisha. Below we report findings on the reasons for smoking shisha. For each meta-theme, the sub-themes identified are summarised in Fig 2, with details provided below.

### Attitudes

Attitudes towards shisha smoking were mainly influenced by flavoured shisha, perceived pleasure from shisha smoking, curiosity about product attributes, perceived health benefits, knowledge of health effects, and regulations that govern shisha in Nigeria.

#### Flavours

Flavour-related characteristics of shisha such as the taste and smell were one of the main reasons for smoking shisha for 29% of the 611 shisha smokers who participated in the quantitative survey. In addition, 65% of the shisha smokers always and 33% sometimes smoked flavoured shisha. Only 2% never smoked flavoured shisha.

During the qualitative interviews, many participants cited the sweetened flavour from shisha as the premise for initiating and continuing to smoke shisha:

*“I started taking shisha at my first shop…*. *I just like the flavour*. *It is the flavour that just pushes me into taking it* …*”* Kano, Female, 33, Low Socio-economic status (SES), Urban

Both the quantitative and qualitative data indicated that shisha was available in a wide selection of attractive flavours. Some qualitative accounts suggested that some shisha smokers had the perception that shisha does not contain any tobacco but it is just a flavour.

*“*… .*I made them understand it’s just the flavour that it’s nothing more*. *When they smell the flavour*, *they were like it’s just fruit*. *It’s not like there is anything there*, *and ever since they are cool with it*.*”* Lagos, Female, 28, High SES, Urban

In the quantitative survey, although most shisha smokers were aware that shisha had tobacco, only 43% thought that shisha contained a significant amount of tobacco.

#### Pleasure

For 28% of the shisha smokers in the quantitative survey, one of the main reasons for smoking shisha was pleasure or fun. 73% of the 611 shisha smokers indicated that when they smoked shisha for the first time they were in a good mood. 75% reported that they loved smoking shisha, whilst 78% reported that it felt good to smoke shisha, 81% liked the motions involved when smoking shisha, and 70% liked holding the shisha pipe.

In most qualitative narratives, shisha smokers revealed that their shisha smoking was influenced by the pleasure which emanated from the shisha smoking itself, the environment where the shisha was smoked, and the company of friends during shisha smoking.

*"…*. *there is this inner joy that it gives you*, *you derive pleasure from taking shisha*, *you know like it puts you in the mood*, *that mood is the sweetest thing that I do derive…… I love taking it with friends when I hang out with friends…*..*It gives you that good mood*, *that sweet mood that you need*. *Even if you are angry*, *when you take shisha it will bring you back to yourself”* Adamawa, Female, 28, Low SES, Rural

*“…Just as I said*, *it’s a party mood*.*…*. *It’s just that party mood……*. *It’s only when we want to extend it to the clubbing level”* Rivers, Female, 31, Low SES, Rural

About 60% of shisha smokers in the quantitative survey initiated and mostly smoked shisha in social settings such as bars, nightclubs, lounges, cafes, restaurants and hotels, usually in the company of friends. In the qualitative interviews, these environments were described as filled with fun and activities such as music and dancing etc. The timing of these activities also meant that shisha was commonly used in the evening, at night or on weekends. In the quantitative survey 90% of shisha smokers mostly smoked it in the evenings. Only a few smokers smoked it occasionally in the morning or day, and during the week when they had to go to work. Only 23% of the 611 shisha smokers reported mostly smoking shisha at home, and this was most common among female shisha smokers, and those from Kaduna and Plateau.

#### Curiosity

20% of the 611 shisha smokers in the quantitative survey cited curiosity about the product as one of the main reasons for smoking shisha. In the qualitative interviews, participants, especially the youth, indicated shisha initiation was often due to curiosity about shisha and the desire to experience first-hand the perceived psychological effects of smoking shisha after having seen others smoking it:

*“*… .*I used to see people take shisha*. *I was amazed by what they did*. *So*, *one day*, *I decided to give it a try to know how taking it feels…*.*”* Gombe, Female, 24, High SES, Urban

Curiosity also emanated from product features such as the flavours as well as the smoke from shisha:

*“What fascinates me about shisha especially is all that smoke*. *I just like the fact that the smoke is heavy*. *Like when you push it out is just heavy and it’s all over the place*. *That’s what fascinates me about it”* Kogi, Female, >30, Low SES, Semi-Urban.

*“Yea*, *you know and the fun of it is when you are pouring out the smoke*, *it’s like people celebrate the smoke*.*”* FCT, Male, 30, High SES, Urban

#### Perceived health benefits of shisha smoking

13% of shisha smokers in the quantitative survey reported that they smoked shisha to help them cope with challenging life situations such as stress, sadness or joblessness. 32% (198/611) screened positive for possible generalised anxiety disorder, and 36% (221/611) screened positive for possible major depressive disorder. About 67% and 66% agreed that shisha calms them down when they are stressed and when they are upset, respectively. This motivation was also mentioned in the qualitative interviews both male and female participants as well as young and older participants:

*"Well*, *I still use it because… some things make somebody depressed*, *and you want to just do anything that will take away that depression or anything negative that you’re thinking about*. *Sometimes you take it for you to just forget your sorrows"*. Gombe, Male, 32, Low SES, Rural

*“Whenever I take shisha it eases my stress*, *I think it’s a positive thing to me*. *When I am depressed or worried about something*, *when I take shisha*, *I will be happy at that moment*, *yes”*. Kogi, Male, >30, High SES, Rural

*“Whenever I am sad and I take shisha*, *it calms me down”*. Kano, Female, 19, Low SES, Urban

*“Let me say I’m kind of unhappy or something*, *when I take it*, *I just have that relaxation of mind*. *So that’s just what I love about it”* Rivers, Male, 34, High SES, Urban

A few (5%) shisha smokers in the quantitative survey reported that they derived physical and neurological benefits from smoking shisha such as strength, agility, feeling high; ability to think, sleep, and keep awake; feeling warm when it is cold; or managing their appetite. This also came up in the qualitative interviews.

*“…*. *it makes me more agile; it gives me morale which I need for the kind of job that I am doing”* FCT, Male, 27, Low SES, Rural

#### Knowledge of health effects

Majority of the current shisha smokers agreed/fully agreed that shisha smoking is extremely dangerous to health (63%). However, only 52% agreed/fully agreed that shisha smoking was ruining their own health. In addition, only 54% of the current shisha smokers had the perception that shisha smoking affects other non-users who are in the vicinity of those smoking shisha. In the qualitative interviews, some participants were able to identify some of the potential health problems from shisha smoking, particularly respiratory problems both for the smokers and non-users in the vicinity. Beliefs that shisha smoking was not harmful to health were partly driven by participants not having experienced adverse health effects associated with shisha that they know of; and shisha flavours which seemed to give the impression that it was safe.

*“For those who say there are health problems*, *that is their opinion*. *As for me*, *there are no health problems associated with using shisha*. *Why I said ‘no’ is because I have been using shisha till today*… . *and to me using shisha does not cause any health problems*. *Currently*, *I do not have any health problems because of using shisha”* Adamawa, Female, 37, High SES, Rural

*“Shisha*, *the flavour*, *the smoke of shisha does not disturb people like cigarettes……”* FCT, Male, 50, High SES, Urban

*“Because the flavour is not harsh*. *The flavour smells nice*. *It will not affect someone that is not taking it*.*”* Kano, Female, 22, Low SES, Urban

A few participants mentioned some remedies that they believed could reduce the negative health effects of shisha smoking:

*“…*.*they tell us you can take soda water*. *They say it will clear the chest*, *it will clear everything”* Plateau, Male, 23, Low SES, Urban

*“Now*, *like [BRAND NAME] contains a little bit of milk*. *You know when you take shisha your head is kind of full upstairs*, *so when you take [BRAND NAME] it calms your head*. *Especially from your chest downward… I don’t know whether it’s the milk inside or the brand in general*, *but I suspect it’s because of the milk inside*. *So*, *it just calms your respiratory or digestive system down”* Kogi, Female, >30, Low SES, Semi-Urban

Most of the current shisha smokers (66%) had the perception that shisha is less harmful than cigarettes. In fact, some participants had stopped smoking cigarettes but continued to smoke shisha for this reason. Only about 24% of current shisha smokers were also current cigarette smokers.

*“Well*, *I stopped smoking cigarettes because it is harmful to the system*. *These smokers are liable to die young*, *I don’t want to die young and I smoke tobacco*, *but I do not smoke cigarettes”* Gombe, Male, 32, Low SES, Rural

#### Regulation

Overall, there was a lack of awareness of the laws and regulations that govern shisha in Nigeria. For the quantitative survey, whilst 39% of shisha smokers were aware of the law or regulations generally regulating tobacco in Nigeria, only about 13% reported knowing any law or regulation on shisha in Nigeria. There was evidence suggesting that the existing tobacco-control laws and regulations are currently not applied to shisha in the same way that they are to other tobacco products. For example, in most cases shisha pots or paraphernalia did not have written or pictorial health warnings as required for tobacco and its products. Only about 16% of shisha smokers reported having noticed any health warnings on shisha packages of shisha tobacco, charcoal, or the water pipe instrument, within the 30 days before the survey. In addition, shisha smoking still happens in places where tobacco smoking is prohibited. All these factors contributed to the perception that shisha was safer than cigarettes.

*“There is no regulation to it [shisha]*. *The only regulation that someone can understand is for instance*, *the federal government wrote something about cigarettes on the pack*, *warning that smokers are liable to die young*.*”* Adamawa, Male, 29, High SES, Semi-Urban

*“I have seen some guidelines about tobacco but I have never seen any guidelines about shisha*. *With tobacco we also see it on the packet and posters that smokers are liable to die young and I have never seen any on shisha*. *That’s what makes me want to continue taking shisha*.*”* Adamawa, Male, 30, High SES, Urban

*“*… ..*it’s [shisha] not offensive to the next person around me that doesn’t take it [shisha]*. *I’ve gone to places where they don’t allow people inside to smoke cigarettes*, *but I was smoking shisha*. *Shisha was allowed but cigarettes were not allowed*. *So that kind of love for it*, *even from people that don’t take it makes me enjoy it more”* Rivers, Male, 34, High SES, Urban

### Subjective norms

We found that peer and social influence were important in the initiation and continuation of shisha smoking. Four main themes under this construct were: social events, friends, family members, and societal views. The majority reported that they typically smoked shisha with other people, and at social events. Only a few participants reported that they typically smoked shisha alone.

#### Social events

As mentioned before, the majority of shisha smokers in the quantitative survey initiated and mostly smoked shisha in social settings. In the qualitative interviews, participants reported that they felt under pressure to join in shisha smoking when they found themselves among a group of shisha smokers at social events or social settings such as bars or nightclubs. They smoked shisha to gain social acceptance or a sense of belonging.

*“What made me start taking shisha was when we went for an occasion in Kaduna*, *we stayed in a hotel all my friends that I went with were using shisha*. *I felt excluded and I decided to join them*.*”* Kano, Male, 35, High SES, Rural

*“I just made up my mind*, *I want to try it*, *let me not be left alone*. *I want to be among them too*.*”* Plateau, Female, 33, High SES, Rural

*“I just continue using it because when I see people using it*, *let them not say that everybody is using it and I’m not*.*”* Gombe, Male, 32, Low SES, Rural

In the quantitative survey 72% of shisha smokers reported that shisha helped people feel more comfortable at celebrations, parties, or in other social gatherings.

#### Friends

20% of the shisha smokers in the quantitative survey reported that shisha smoking by friends was one of the main reasons for smoking shisha. Almost all shisha smokers (97%) in the quantitative survey reported having shisha smokers among their closest friends. 34% had seen or heard about shisha for the first time from a friend, 94% were with at least one friend when they first smoked shisha, and 88% usually smoked shisha with at least one friend. The qualitative interviews also echoed these findings:

*“These things about peer group influence where you hang out with friends*, *you can go to 1 or 2 or 3 occasions*. *Before you know it*, *this one [shisha] is on the table*. *When you see others taking it*, *you like to now engage to see what is in these things so that was how I started”* Anambra, Male, 32, High SES, Urban

*“I have a friend and we went to…and I started taking shisha*. *We went there and I saw her taking shisha and from there she influenced me and little by little I continue”* Kano, Female, 19, Low SES, Urban

*“We went for an occasion as I usually go out for parties and occasions*. *At one of the numerous occasions was when she [my close friend] finally introduced me to taking shisha"* Gombe, Female, 39, High SES, Urban

Some respondents reported having friends who were not shisha smokers but viewed their shisha smoking favourably, and this was attributed to the flavours:

*“I have some friends that don’t take shisha*. *When I sit down with them*, *maybe I start taking that shisha*. *Some of them even recommend the flavour*. *They say they like this and this flavour*. *I will say okay*, *can you take this*? *They will say no*, *no*, *no*. *But they like my smoking in their presence*.*”* FCT, Male, 45, High SES, Urban

#### Family members

Only 1% of the 611 shisha smokers who participated in the quantitative survey mentioned shisha smoking by family members as the main reason for shisha smoking. However, 30% reported having family members who were current shisha smokers. For some participants, initiation and continuation of shisha smoking was influenced by family members who were shisha smokers and approved of the participants’ shisha smoking:

*“When my older sister came back from [COUNTRY NAME]*, *she used to take shisha in our room and eventually she taught me*, *and I began to take shisha”* Kano, Female, 23, Low SES, Urban

*“All my uncles do take shisha*. *I usually see them taking shisha*. *So*, *when my friends came and introduced me*, *I said let me just join them”* Plateau, Male, 35, Low SES, Urban

However, there were some contrary reports of family members not being supportive of shisha, which resulted in the concealment of shisha smoking from family members:

*“None of my relatives knows that I do take shisha*, *but my friends know"*. Oyo, Male, 34, High SES, Urban

*“…*.*my people don’t even know I take shisha*, *at least for my parents*, *I don’t want to disappoint them*. *You know parents when they see you will say you have joined bad gangs*. *So*, *when they respect you*, *you’ve to respect them"* Kano, Female, 33, Low SES, Urban.

#### Societal views

The majority of shisha smokers agreed/ strongly agreed with the statements that shisha is cool and trendy (88%), shisha smoking is gaining popularity (84%), and that stylish persons smoke shisha (76%). However, data from the qualitative interviews portrayed a consensus among participants that smoking was generally not socially accepted. The low tolerance emanated from religious beliefs as well as perceived health risks. People who smoked were perceived as being irresponsible, amoral or having perverse behaviour. Notably, however, society’s low tolerance was not a deterrent to shisha smoking. Rather, shisha smokers adopted a means of living a dual lifestyle to mask their smoking habit, preferring to smoke at organised social spots like clubhouses, bars, and lounges.

*"A lot of people look at people that take shisha differently*. *They say this one is a bad person*, *bad girl"* Plateau, Female, 33, High SES, Rural

*"They look at me with bad eyes*, *as a bad guy*, *like you are so arrogant or something like that*. *But my parents won’t look at me anyhow because they do not know*, *and I won’t do it in front of them"* Kaduna, Male, 31, Low SES, Urban

*“Nobody likes all these things especially if they see you taking this thing [shisha]*, *they may think that maybe you are taking weed or maybe you will create a bad image for their children or something like that*. *So*, *most of the time I take it inside my room or bedroom*.*”* Gombe, Female, 32, High SES, Urban

The negative societal views about shisha smoking were perceived as even more pronounced towards females when compared to male shisha smokers.

*“People around the community dislike seeing a woman use shisha*. *So that is why I use my shisha at home where people will not see me or scold me”* Oyo, Female, 29, High SES, Rural

*“……*. *If it is a female that takes shisha*, *people will say she is wayward or call her a woman of no honour or other negative words*.*”* Kano, Female, 19, Low SES, Urban

When asked questions comparing shisha to cigarettes, 79% of the quantitative survey shisha smokers agreed/strongly agreed that shisha smoking is more socially acceptable than cigarette smoking, and 76% agreed/strongly agreed that shisha smoke is more accepted by society than cigarette smoke. In addition, 92% agreed/ strongly agreed that females were more comfortable with smoking shisha compared to cigarettes.

### Behavioural control

Behavioural control was influenced by: 1) perceived ease or difficulty in smoking shisha which mainly stemmed from the acute side effects from smoking shisha, availability and cost; and 2) perceived ease or difficulty in quitting shisha smoking.

#### Perceived ease or difficult in smoking shisha: Acute side effects from smoking shisha

About 44% of participants reported experiencing negative effects when they first started smoking shisha. A similar percentage also reported side-effects with continued use. These included choking, headache, chest pain, loss of consciousness, vomiting and passage of frequent stools. However, many qualitative interview respondents reported being able to overcome these initial challenges to become proficient at smoking shisha through perseverance and encouragement from significant others so that smoking shisha became an enjoyable experience:

*“The first time I took it I just started coughing*. *So*, *they [friends] said I should not see it that way*, *that I should still try it*. *They said I should relax my mind and just do it*. *That was it****”*** Rivers, Female, 25, Low SES, Rural

*"The first time I smoked shisha*, *it gave me a headache*, *it disturbed me that day*. *But later we now noticed that it is because there was no mixing in that shisha*. *When we started mixing the shisha well*, *we saw that it was different…*..*"* Kaduna, Male, 31, Low SES, Urban

#### Perceived ease or difficult in smoking shisha: Availability

In the quantitative survey, only 1% of shisha smokers reported availability as one of the main reasons for smoking shisha. However, from the qualitative interviews, there was an overall perception that shisha was easily available, including to children. This was mainly attributed to the lack of laws and regulations that control the sale and use of shisha:

*“These substances [shisha] are all over the place*. *I wish the government could control them*, *and be more active in controlling the use*, *because young lads*, *even lads that are not aged 18 yet are already sucked in this activity*. *I wish they would be more conscious about it*. *I believe there is no control in my community”* Oyo, Male, 33, High SES, Urban

*“From the World Health Organization or government*, *the usage of tobacco is from 18 years and above*. *I see some boys of 14–15 years taking shisha and nobody talks to them*. *Nobody educates them on the effect”*. Ebonyi, Male, 34, Low SES, Urban

25% of shisha smokers in the quantitative survey usually bought their shisha from clubs, bars, lounges, hotels, cafés or restaurants, 18% mentioned convenience stores, mini-markets or produce markets, 16% mentioned smoke shops or tobacco specialty stores, and 15% mentioned supermarkets.

#### Perceived ease or difficult in smoking shisha: Cost

The cost of shisha seemed to be prohibitive for shisha smoking. In the quantitative survey, none of the respondents mentioned the cost/ affordability as one of the reasons for smoking shisha, and 52% agreed/fully agreed that they spend too much money on shisha. Qualitative interview respondents mentioned that the cost of shisha was higher than that of cigarettes. This was viewed as a deterrent, particularly for those from lower socio-economic classes:

*"Let me tell you something about using shisha and cigarettes*. *There’s quite a difference*. *Even class*. *There are classes of people in society you won’t see with a cigarette*, *but they take shisha*. *So*, *cigarettes everybody can afford*. *Ten-naira*, *twenty-naira*, *fifty Naira*. *But not shisha*, *you can’t buy shisha with fifty Naira*, *or twenty Naira or hundred Naira*. *It costs money"* Adamawa, Male, 45, High SES, Urban

It is possible that this has an influence on the frequency of use: only 14% of shisha smokers were daily users, and 64% reported typically having just one shisha session on the days they smoked shisha. However, 40% reported that a typical shisha session was more than an hour long, and 57% consumed between 2–5 bowls of shisha in a typical smoking session. Participants also revealed strategies that they used to lower the costs, for example, owning paraphernalia, which meant only having to buy the flavours and the coals:

*"I do spend almost 3000 Naira to 4000 Naira on the flavours and coals in a week because I have my pot already*. *I buy the flavours and the stuff*, *just once or twice*. *When I go to the shop*, *I make sure I buy everything I need*, *all that will last me for the week*. *It’s of 3 categories*, *you get 800 Naira*, *1200 and there is that of 700 too…… I usually go for that 1200*. *The coal is just 300 Naira*.*"* Plateau, Female, 33, High SES, Rural

Quite commonly, acquaintances also shared the cost of shisha at public locations, a pattern that echoes in both rural and urban locations alike.

#### Perceived ease or difficulty in quitting shisha smoking

50% of the shisha smokers in the quantitative survey believed that they could choose to quit smoking shisha at any time with little difficulty. However, 241 of the 611 shisha smokers (i.e., 40%) had attempted to stop smoking shisha during the past 12 months preceding the study without success. 54% of the 241 who had tried to stop smoking shisha cited personal health concerns as the main reason, with the next most-cited reason being wanting to set a good example for children (20%).

When asked about their intention to quit, 50% of the 611 shisha smokers were not interested in quitting at all, whilst 30% indicated that they would quit someday but not in the next 12 months. Only 13% were thinking of quitting within the next 12 months.

In the qualitative interviews, several participants indicated that they wanted to quit shisha but it was a habit that was difficult to stop:

*"If I tell you that there is any benefit I derive from taking shisha*, *I am lying to you*. *It has just formed part of my life and it is now difficult to stop"*. Gombe, Male, 24, Low SES, Rural

*“…*.. *it doesn’t have any positive impact in your life*, *it’s just a bad habit you started and the ability to stop it is not there*. *It becomes a part of you until you can work on yourself"* FCT, Male, 30, High SES, Urban

In the quantitative survey, 64% of shisha smokers said shisha smoking is addictive. However, 78% believed that cigarettes were much more addictive than shisha, while 15% believed that shisha was much more addictive than cigarettes.

190 (i.e., ∼80%) of the 241 shisha smokers who had tried to stop had tried without any help. 7.5% had received cessation counselling, 3.4% had used nicotine replacement therapy, 4.6% indicated using other prescription medications, 5.6% had used traditional medicines and 2.5% had used a quit-line or telephone support. Some shisha smokers highlighted the potential role of significant others in supporting shisha smokers to reduce or quit smoking:

*“Well*, *my mother scolded me and instructed me to stop taking shisha*. *I promised her I would stop*. *I have gradually reduced my intake of shisha*.*”* Kano, Female, 19, Low SES, Urban

*"She (sister) didn’t tell me why I should stop taking shisha*. *I asked why but she just instructed me to stop*. *I replied to her that “you know that will be a gradual process” and I have reduced the smoking of shisha*.*"* Kano, Female, 23, Low SES, Urban

Ability to resist smoking shisha in different situations was low. For each of the following situations, only about 40% were absolutely sure that they would be able to refrain from shisha smoking: when feeling nervous, depressed, angry, very anxious, feeling the urge to smoke, thinking about a difficult situation, having a drink with friends, in the presence of other smokers, celebrating something, or when drinking alcohol.

## Discussion

### Summary of key findings

In our study, the most common reasons for smoking shisha include flavours, perceived pleasure from shisha smoking, curiosity about product attributes, beliefs about health benefits, limited knowledge on the health effects, the availability of and ability to smoke shisha in many places, the need for social acceptance at events and by friends, having friends and family members who smoke shisha, and poor enforcement and compliance monitoring of the existing tobacco control laws. The high cost of shisha and negative societal views on shisha smoking were perceived as prohibitive. The ability to resist smoking shisha in different situations was low. The study findings also suggest that quitting shisha smoking without support is difficult. The data from the qualitative interviews and quantitative survey were overly either convergent or complementary.

In line with findings from other studies on flavoured tobacco products [50,51], we found that in Nigeria, shisha flavours made people want to try or continue using shisha, provided variety, and gave both smokers and non-smokers the impression that shisha is safe, or safer than cigarettes. Flavours also contributed to the perception among some shisha smokers and non-smokers that it was not tobacco. In another study in Nigeria, 25% of shisha smokers also cited flavours as one of the main reasons for smoking shisha [6]. Although most of our respondents self-reported that they did not smoke cigarettes, other studies have found that flavoured tobacco products are more appealing than non-flavoured products, particularly to young people who may start with these products and progress to long-term tobacco use [52–54]. There have also been reports that the flavours give the smoker a sensation that is beyond what they get from non-flavoured products [50,55]. Several countries prohibit flavours, although, for some of these countries, the tobacco products covered are restricted and do not often include shisha [56]. However, for some countries, including Ethiopia, Niger, Senegal and Uganda in Africa flavour restrictions cover all products manufactured or derived partly or entirely from tobacco leaf [56]. This is consistent with the WHO FCTC recommendation to either ban or restrict the use of flavours in tobacco products [13]. We also found that other product characteristics such as the smoke produced by shisha also had a part to play in the initiation and continued use of shisha.

Misconceptions about the health hazards associated with shisha are well documented in the literature [4–6,50,51,57]. In our study, in addition to flavours, one of the key underlying factors that contributed to the misconception that shisha was safe and led to positive evaluations of shisha was weak regulation, including lack of enforcement and compliance monitoring. For example, most shisha products did not have written and graphical health warnings as mandated by Nigeria’s tobacco control laws [14,15]. The adoption of health warnings on shisha is a cost-effective strategy for reducing its use [13]. A study in Egypt to understand the impact of pictorial health warnings on shisha reported that 58.5% of waterpipe smokers were motivated to think about quitting; 64.5% reduced their consumption; 42.2% forgo a smoke; 24.5% attempted to quit [58]. They also motivated 57.1% of former waterpipe smokers to successfully quit, and 59.3% of non-smokers to remain smoke-free [58]. Health warnings on cigarettes have also been shown to stimulate smoking cessation responses in smokers and prevent initiation in non-smokers. The fact that some shisha smokers had the perception that shisha did not contain tobacco but only flavours is worrisome and is also partly attributable to the lack of compliance monitoring and enforcement of tobacco control laws on shisha as done with other tobacco products.

In Nigeria, tobacco smoking in indoor and outdoor public spaces for the service of consumption of food/drink including cafeterias, restaurants, and any other place for public refreshment and hospitality is prohibited, except in designated smoking areas which should be 10 metres away from the entrance [14,15]. The tobacco control laws also mandate no smoking signs (both in writing and graphical) at the main entrance and other entrances of such establishments. However, from our findings, shisha seems to be readily available, and its use mainly occurs in bars, nightclubs, and hotels. This could be because these establishments are not explicitly named in the law and are assumed not to be covered. As indicated by one of our respondents, even some places that prohibit cigarette smoking allow shisha smoking, which added to the perception that shisha was safer than cigarettes. Other studies have also reported that many public shisha smoking places continue to open across Nigeria despite the tobacco control laws that also cover shisha [6].

The high cost could be prohibitive to shisha smoking, as it is for other tobacco products. In Nigeria, a specific tax applies to all smokeless tobacco products and shisha, at ₦1,000 ($ 2.6) per kg or ₦3,000 ($7.8) per litre [59]. Such policies have to consider that shisha smoking is predominantly a group behaviour where costs could be shared. In addition, the use of own paraphernalia to reduce the costs also needs to be accounted for. A unit package of shisha doesn’t seem to be as well defined as it is for cigarettes, which might hinder such policies.

We found that subjective norms play a significant role in the initiation and continuation of shisha smoking: use is mostly a communal practice where initiation and continued use occur in social group settings. Initiation was reported as occurring in the presence of significant others such as friends and relatives in public places like bars, clubs, and restaurants or at social events such as parties as well as at homes of friends. Other researchers and policymakers have raised awareness about the challenges presented by the shared shisha smoking experience, particularly around infectious disease transmission [60]. Whilst respondents in this study perceived that shisha smoking, particularly by women, was viewed negatively by society, in other societies outside Nigeria shisha smoking among both men and women is acceptable, particularly where it is believed to be rooted in public culture and tradition [53,61]. Negative societal views may protect non-smokers from initiating smoking, and deter smokers, particularly women, from smoking shisha in public places. In this study, many of our women participants conformed to societal expectations in public but smoked shisha privately in the homes or in safe spaces such as among closely knitted set of friends where there was less likelihood of stigmatisation. This might mean that shisha smoking is underreported. Nevertheless, our findings also suggest that family members and society can be a resource for discouraging shisha smoking. This means that interventions might also need to be directed at communities instead of just at individuals.

Participants suggested that smoking shisha improved their mood, reduced stress, and resulted in pleasurable experiences. These findings have also been documented by other researchers [24,57,62]. However, evidence has shown a significant positive association between tobacco smoking and mental health problems such as anxiety, tension and depression [63,64]. On the other hand, stopping smoking is associated with lower levels of anxiety, stress and depression, improved mood and quality of life [65]. Thus, our findings emphasise the importance of promoting safe ways of maintaining emotional and mental well-being. Our study also indicates that self-efficacy to stop using shisha is low among smokers: they need support to stop smoking shisha.

### Strengths and limitations

We used both quantitative and qualitative in-depth interviews to understand the factors that influence shisha smoking in Nigeria. This means that we were able to triangulate the findings from these two sources, and improve the robustness of our conclusions. There was either convergence or complementarity between the two datasets for almost all themes. The exceptions were: 1) “societal views” where the quantitative results suggested that shisha smoking was acceptable whilst the qualitative findings contradicted this; and 2) “availability” which was cited as one of the main reasons for smoking shisha by only 1% in the quantitative data, whilst there was an overall perception that shisha was easily available, including to children, in the qualitative data.

For the qualitative interviews, we were able to reach data saturation in terms of scope and replicability [28], and, as such, the study findings would be transferable to other similar populations. For the quantitative survey we recruited participants from 10 randomly selected EAs in the 12 most urbanised states covering all the six geographical zones of Nigeria to enhance the generalizability of our study findings. The sample frame was limited to those with phone numbers, which might restrict the applicability of the findings to the general population in Nigeria. However, we were able to translate our data collection tools into local languages and also administer the questionnaire and interviews in local languages for those who preferred this which also enhanced generalizability.

### Policy and practice recommendations

Considering their influence on the initiation and continued use of shisha, restrictions on flavours have the potential to minimise initiation and use, including among young people, and to protect the general public as a whole in Nigeria (13). In addition, compliance monitoring and enforcement of the existing tobacco control laws need to be strengthened, particularly in the following areas: 1) smoke-free environments in indoor and outdoor places where shisha smoking commonly occurs such as bars, nightclubs and hotels including the presence of no smoking signage; 2) health (both written and graphical) warnings in English on shisha products including the pots; 3) application of the specific tax; and 4) sale of shisha to minors. It is also imperative that as part of tobacco control, there is awareness raising on the harmful effects of shisha smoking on health, including mental health and emotional well-being, and how these harmful effects outweigh any temporary perceived stress relief. There is also a need to provide interventions to help people stop smoking shisha, and advocate for safer ways to improve and maintain good emotional and mental well-being such as regular exercise. These initiatives could leverage community support, particularly in those communities where there are negative views towards shisha smoking.

### Research recommendations

Future research could examine the extent of compliance with tobacco control laws concerning shisha and develop and test different strategies to monitor and improve compliance and enforcement. There is also a need to develop and evaluate the effectiveness of educational campaigns, removing flavours and interventions to help shisha smokers quit. Researchers also need to monitor the impact of new policies as and when they are developed.

## Conclusions

For shisha smokers in Nigeria, the positive attitudes towards shisha smoking are influenced by shisha flavours, perceived pleasure from shisha smoking, curiosity about product attributes, beliefs about health benefits, limited knowledge on the health effects, and weak regulation. Having friends and family members who smoke shisha and the need to belong, particularly during social events, also promote shisha smoking. On the other hand, negative societal views towards shisha smoking are potentially a protective factor. The availability of and ability to smoke shisha in many places makes shisha more accessible, whilst the high costs of shisha are potentially prohibitive. The findings also indicate that quitting shisha smoking without support is difficult. This information can be used to formulate policy measures and health interventions that can reduce the prevalence of shisha smoking in Nigeria.

## Supporting information

S1 Checklist Inclusivity in global research questionnaire. (DOCX)

S1 Fig Urbanization rates by state. (DOCX)

S1 Text Nigeria shisha survey questionnaire– 2022. (DOCX)

S2 Text Interview guide. (DOCX)

S1 Table Triangulation matrix. (XLSX)
