# Supplementary material for: Reasons for shisha smoking: Findings from a mixed methods study among adult shisha smokers in Nigeria
Source: PLOS Glob Public Health. 2024 Feb 2;4(2):e0002853. doi: 10.1371/journal.pgph.0002853 (PMC10836660; doi:10.1371/journal.pgph.0002853)
Supplement: S1 Fig — (DOCX) [file pgph.0002853.s002.docx]

**Reasons for shisha smoking: findings from a mixed methods study among adult shisha smokers in Nigeria**

**S1 Fig.** **Urbanization rates by state**


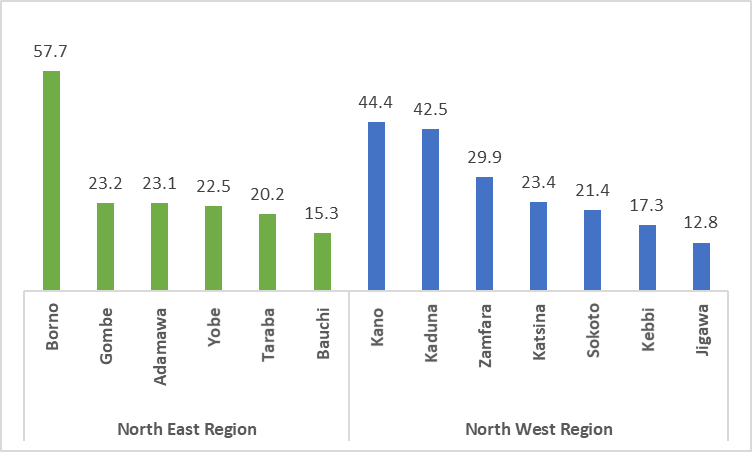


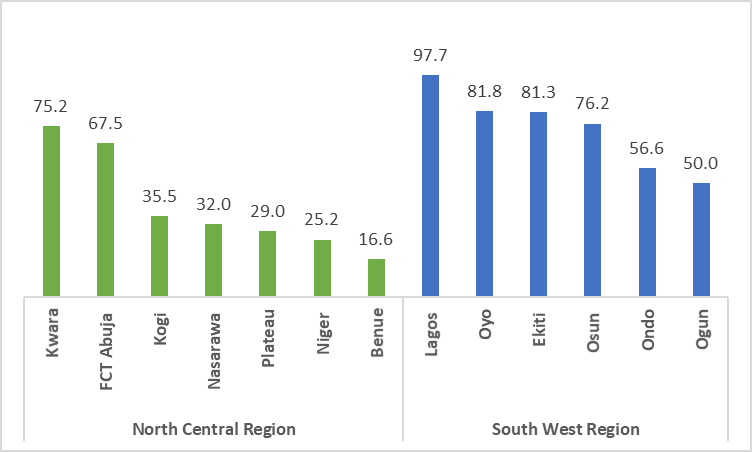


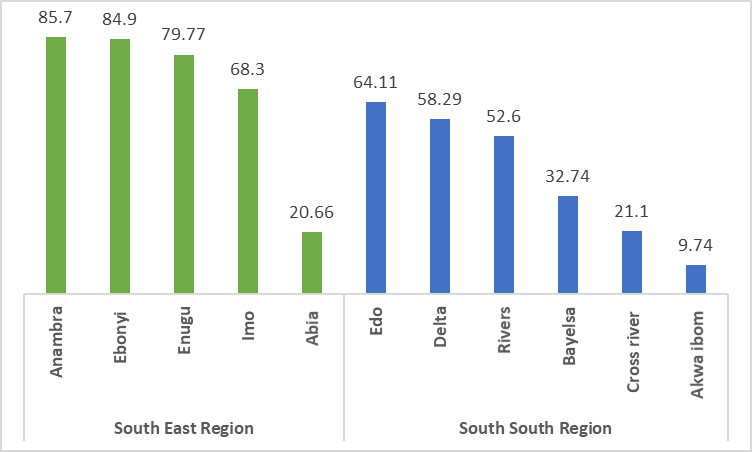


**Source:** Nigeria Demographic and Health Survey (NDHS, 2018)
