# Supplementary material for: Reasons for shisha smoking: Findings from a mixed methods study among adult shisha smokers in Nigeria
Source: PLOS Glob Public Health. 2024 Feb 2;4(2):e0002853. doi: 10.1371/journal.pgph.0002853 (PMC10836660; doi:10.1371/journal.pgph.0002853)
Supplement: S1 Text — (DOCX) [file pgph.0002853.s003.docx]

**Reasons for shisha smoking: findings from a mixed methods study among adult shisha smokers in Nigeria**

**NIGERIA SHISHA SURVEY QUESTIONNAIRE-2022**

Contents

[SECTION 1: DEMOGRAPHIC AND SOCIO-ECONOMIC CHARACTERISTICS 2](#_Toc101791172)

[SECTION 2: SHISHA USE BEHAVIOUR AND PATTERNS 5](#_Toc101791173)

[SECTION 3: REASONS FOR USE OF SHISHA *(CURRENT OR PAST USERS ONLY)* 15](#_Toc101791174)

[SECTION 4: ATTITUDE AND PERCEPTION TOWARDS SHISHA USE (CURRENT AND PAST USERS ONLY) 16](#_Toc101791175)

[SECTION 5: CAPACITY TO DISCONTINUE SHISHA USE 17](#_Toc101791176)

[SECTION 6: KNOWLEDGE AND HEALTH PERCEPTIONS OF SHISHA USE 18](#_Toc101791177)

[Section 7: STRESS, ANXIETY AND DEPRESSION 19](#_Toc101791178)

[SECTION 8: MEDIA AND KNOWLEDGE OF REGULATION OF SHISHA USE IN NIGERIA 20](#_Toc101791179)

[SECTION 9: AIRTIME NUMBER AND CONTACT INFORMATION 21](#_Toc101791180)

[APPENDIX A: SHISHA SURVEY AND THEORY OF PLANNED BEHAVIOUR (TPB) MAPPING 22](#_Toc101791181)

# SECTION 1: DEMOGRAPHIC AND SOCIO-ECONOMIC CHARACTERISTICS

| NO. | QUESTIONS AND FILTERS | | CODING CATEGORY | | | | | | SKIP |
| --- | --- | --- | --- | --- | --- | --- | --- | --- | --- |
|  | Respondent ID | | \|____\| | | | | | |  |
|  | Interview Date: | | Day: \|____\|____\|  Month: \|____\|____\|  Year: **\|_**2_\|_0_\|_2_\|_2**\|** | | | | | |  |
|  | Start Time (24 Hour clock) | | Hour: \|____\|____\|  Min: \|____\|____\| | | | | | |  |
|  | End Time (24 Hour clock) | | Hour: \|____\|____\|  Min: \|____\|____\| | | | | | |  |
|  | Language of Interview | | 1. English 2. Igbo 3. Hausa 4. Yoruba 5. Pidgin English | | | | | |  |
|  | | | | | | | | | |
| 100 | I am going to first ask you about your background | | | | | | | | |
|  | In what month were you born? | 01. . . . . . . . . . . . . . . . .. . . ... . . . . . . . .. . . 1  02. . . . . . . . . . . . . . . . . . . . . . . . . . . . . . . . 2  03. . . . . . . . . . . . . . . ... . . . . ... . . . .. . . . . . 3  04. . . . . . . . . . . . . ... . . . . . . . .. . . . . . . . . . 4  05. . . . . . . . . . . . . . . . . . . . . .. . . . . .. . . . . 5  06 . . . . . . . . . . . . . ... . . . . . . . . . . . . . . . . 6  07 . . . . . . . . . . . . . . . . . . . . . . . .. .. . .. . . . 7  08 . . . . . . . . . . . ... . . . . . . . . . . . . . . . . . . 8  09 . . . . . . . . . . . . . . . . . . . . . . . . . . . . . . . 9  10. . . . . . . . . . . . .. . . . . . . . .. . . .. . . . . . 10  11. . . . . . . . . . . . .. . . . . . . . . ... . . . . . . . . 11  12. . . . . . . . . . . . . . . . . . . . . . . .. . . .. . . . 12  DON’T KNOW. . . . . . . . . . . . . . . . . . . .. 98 REFUSED . . . . . . . . . . . . . . . . … . . . . . 99 | | | | | | |  |
| 101 | In what year were you born? |  | | | | | | |  |
| 102 | How old were you at your last birthday?  Compare and Correct 105 and/or 106 If  Inconsistent. | AGE IN COMPLETED  YEARS | | | | | | |  |
| 103 | What is your gender? (Only ask if necessary) | Male -------------------------------- 1  Female-------------------------------2  Other (specify) ---------------------9 | | | | | | |  |
| 104 | In which **State** are you currently living? |  | | | | | | |  |
| 105 | In which **Local Government Area (LGA)** are you currently living? |  | | | | | | |  |
| 106 | Location  (According to definition of LGA along urban and rural by NBS in the sample frame)  (To be populated based on response to Q105 | Urban --------------------------------------1  Rural ---------------------------------------2 | | | | | | |  |
| 107 | What is the highest level of education you have completed? | No Formal Schooling------------------------ 1  Less Than Primary School Completed-----2  Primary School Completed ----------------- 3  Junior Secondary School Completed ----- 4  Senior Secondary School Completed ----- 5  Less than College/University Degree Completed ------------------------------------- 6  College/University Degree Completed-----7  Post-Graduate Degree Completed ----------8  None -------------------------------------------95  Don’t know ---------------------------------- 99  Refused -------------------------------------- 98 | | | | | | |  |
| 108 | What is your current marital status? | Single-------------------------------------------1  Married-----------------------------------------2  Separated --------------------------------------3  Divorced --------------------------------------4  Widowed --------------------------------------5  Refused ---------------------------------------95 | | | | | | |  |
| 109 | What is your ethnicity? | Ekoi -------------------------------------------1  Fulani------------------------------------------2  Hausa -----------------------------------------3  Ibibio -----------------------------------------4  Igala ------------------------------------------5  Igbo -------------------------------------------6  Ijaw/Izon -------------------------------------7  Kanuri/Beriberi ------------------------------8  Tiv ---------------------------------------------9  Yoruba ---------------------------------------10  Others (Specify) ----------------------------96  Don’t Know -------------------------------- 99 | | | | | | |  |
| 110 | What is your religion? | Christianity------------------------------------1  Islam-------------------------------------------2  Traditional-------------------------------------3  None-------------------------------------------95  Don’t know-----------------------------------98  Others (specify) -----------------------------96  Refused ---------------------------------------99 | | | | | | |  |
| 111 | Which of the following best describes your **“main”** work status over the past 12 months ? Government employee, Non-government employee, self-employed, student, homemaker, retired, unemployed -able to work, unemployed- unable to work?  *[Include Subsistence Farming as Self-Employed]* | Government employee …………………..1  Non-government employee------ -----------2  Self-employed --------------------------------3  Student……………………………..…….4  Housewife………………………….…….5  Retired…………………….……………..6  Unemployed, able to work……………….7  Unemployed, unable to work…………….8  Don’t know…………….……………..…98  Refused …………………………………99 | | | | | | |  |
| 112 | Please tell me whether you or your household has the following items:  Note: **Response categories**  Yes ---1  No ---2  Don’t Know --- 99  Refused ---95 |  | | Yes | No | | Don’t  Know | Refused |  |
|  |  | Electricity  Flush toilet  Fixed Telephone  Cell telephone  Television  Radio  Refrigerator  Car  Moped/scooter/motorcycle  Washing machine  Engine boat  Horse/camel/donkey | |  |  |  | |  |  |

# SECTION 2:  SHISHA USE BEHAVIOUR AND PATTERNS

| 200 | I would now want to ask you some questions about shisha use | | | | | | | |
| --- | --- | --- | --- | --- | --- | --- | --- | --- |
|  | **ALL RESPONDENTS** | | | | | | | |
| 201 | Have you ever smoked shisha even one or two puffs? | | Yes------------------------------------------------------1  No-------------------------------------------------------2 → | | | | 243 | |
|  | **EVER USERS (CURRENT AND PAST)** | | | | | |  | |
| 202 | Do you currently use shisha on a daily basis, less than daily or not at all? | | Daily ----------------------------------------------------1→  Less than daily ----------------------------------------2→  Not at all ----------------------------------------------3 | | | | 209  209 | |
| 203 | In the past have you used shisha on a daily basis or less than daily? | | Daily ---------------------------------------------------1  Less than daily ---------------------------------------2 | | | |  | |
|  | **PAST USERS ONLY** | | | | | |  | |
| 204 | How long has it been since you stopped smoking shisha? | | Years -------------------------------------------------------1  Months -----------------------------------------------------2  Weeks ------------------------------------------------------3  Don’t know -----------------------------------------------8 | | | |  | |
| 205 | Enter number of (years, months, weeks) | | Record here   \|  \|  \|  \| \| --- \| --- \| --- \| | | | |  | |
| 206 | Which of the following influenced your decision to stop smoking shisha?  *(Tick as apply)*  *(Multiple responses allowed)* | | Personal Health concerns ------------------------------A  Costs ------------------------------------------------------B  Family pressure or disapproval -----------------------C  Smoking restrictions -----------------------------------D  Friends’ disapproval ------------------------------------E  Concern that your smoking is bad for others --------F  Wanting to set a good example for children? -------G  Others (Specify) ---------------------------------------X  Don’t know ----------------------------------------------Y  Refused ----------------------------------------------- Z | | | |  | |
| 207 | Did you receive help or advice before you stop using shisha? | | Yes -------------------------------------------------------1  No --------------------------------------------------------2  Not sure -------------------------------------------------9 | | | |  | |
| 208 | From whom did you receive this help or advice from?  *(Multiple responses allowed)* | | Family Member ------------------------------------------A  Health Programme --------------------------------------B  Health Professiona l-------------------------------------C  Friend -----------------------------------------------------D  Religious Grounds --------------------------------------E  Others (Specify) ----------------------------------------X | | | |  | |
|  | **EVER USERS (CURRENT AND PAST USERS)** | | | | | |  | |
| 209 | How old were you when you FIRST tried smoking shisha, even once? | | \|  \|  \| \| --- \| --- \|   Enter 98, if don’t know;  Enter 99, if refused. | | | |  | |
| 210 | How many years ago did you FIRST try smoking shisha, even once? | | \|  \|  \| \| --- \| --- \| | | | |  | |
| 211 | Where did you FIRST see or hear about shisha? | | From a friend --------------------------------------------1  From the news -------------------------------------------2  At the bar/club -------------------------------------------3  From a family member ---------------------------------4  Music Videos -------------------------------------------5  Social event (i.e. parties) ------------------------------6  Social media (FB, Instagram etc.) -------------------7  Others (Specify) ---------------------------------------8 | | | |  | |
| 212 | Where were you when you FIRST used shisha? | | In a  café/restaurant-/bar/club ---------------------1  Bar/club ----------------------------------------------2  In my own home ------------------------------------3  At a family member’s home ----------------------4  At a friend or acquaintance house ----------------5  At a social event (i.e. parties) ---------------------6  Others  (specify) ------------------------------------7 | | | |  | |
| 213 | Who were you with when you FIRST used shisha?  (Multiple responses allowed) | | Alone -------------------------------------------------A  With one friend -------------------------------------B  With more than one friend ------------------------C  With a family member -----------------------------D  With more than one family member --------------E  With a new acquaintance ---------------------------F  Others (specify) -------------------------------------X | | | |  | |
| 214 | Where any of these moods influence your use of shisha right before you FIRST used shisha? | | Happy --------------------------------------------------1  Feeling depressed ------------------------------------2  Anxiety ------------------------------------------------3  Stressed ------------------------------------------------4  Sad ------------------------------------------------------5  Other (specify) ----------------------------------------6 | | | |  | |
| 215 | Did you feel any discomfort the FIRST time you used shisha? | | Yes -------------------------------------------------1  No -------------------------------------------------2 → | | | | 217 | |
| 216 | What type of discomfort did you feel when you FIRST used shisha? | | Coughing ------------------------------------------------1  Choking -------------------------------------------------2  Light-headedness --------------------------------------3  Headache -----------------------------------------------4  Vomiting/nausea ---------------------------------------5  Chest pain -----------------------------------------------6  Bitter taste ----------------------------------------------7  Loss of consciousness ---------------------------------8  Frequent stooling --------------------------------------9  Others (Specify) ---------------------------------------96  Don’t know --------------------------------------------98  Refused -------------------------------------------------99 | | | |  | |
| 217 | Where do you usually buy your shisha? | | Supermarket -------------------------------------------1  Convenience store/mini market/produce market--2  Smoke shop or tobacco specialty store ------------3  Café/Restaurant ---------------------------------------4  Friends or relative ------------------------------------5  Internet -------------------------------------------------6  I never buy shisha products -------------------------7  Abroad -------------------------------------------------8  Other (specify) --------------------------------------------9 | | | |  | |
| 218 | What is your usual place for smoking shisha?  **READ OUT Options of each response category:** | |  | | | |  | |
| a | In a café/restaurant | | Almost always/always---------------------------------1  Usually/most of the time------------------------------2  Often-----------------------------------------------------3  Sometimes----------------------------------------------4  Seldom/rarely------------------------------------------5  Never---------------------------------------------------6  Don’t know/don’t remember------------------------9 | | | |  | |
| b | In own home | | Almost always/always---------------------------------1  Usually/most of the time------------------------------2  Often-----------------------------------------------------3  Sometimes----------------------------------------------4  Seldom/rarely------------------------------------------5  Never---------------------------------------------------6  Don’t know/don’t remember------------------------9 | | | |  | |
| c | In someone else’s home | | Almost always/always---------------------------------1  Usually/most of the time------------------------------2  Often-----------------------------------------------------3  Sometimes----------------------------------------------4  Seldom/rarely------------------------------------------5  Never---------------------------------------------------6  Don’t know/don’t remember------------------------9 | | | |  | |
| d | University accommodation | | Almost always/always---------------------------------1  Usually/most of the time------------------------------2  Often-----------------------------------------------------3  Sometimes----------------------------------------------4  Seldom/rarely------------------------------------------5  Never---------------------------------------------------6  Don’t know/don’t remember------------------------9 | | | |  | |
| e | In other place | | Almost always/always---------------------------------1  Usually/most of the time------------------------------2  Often-----------------------------------------------------3  Sometimes----------------------------------------------4  Seldom/rarely------------------------------------------5  Never----------------------------------------------------6  Don’t know/don’t remember-------------------------9 | | | |  | |
| 219 | Who do you usually smoke shisha with? | |  | | | |  | |
| a | Alone  *Please check ONLY ONE* | | Almost always/always ----------------------------1  Usually/most of the time -------------------------2  Often ------------------------------------------------3  Sometimes -----------------------------------------4  Seldom/rarely -------------------------------------5  Never -----------------------------------------------6  Don’t know/don’t remember --------------------7 | | | |  | |
| b | With one friend  *Please check ONLY ONE* | | Almost always/always ----------------------------1  Usually/most of the time -------------------------2  Often ------------------------------------------------3  Sometimes -----------------------------------------4  Seldom/rarely -------------------------------------5  Never -----------------------------------------------6  Don’t know/don’t remember --------------------7 | | | |  | |
| c | With more than one friend  *Please check ONLY ONE* | | Almost always/always ----------------------------1  Usually/most of the time -------------------------2  Often ------------------------------------------------3  Sometimes -----------------------------------------4  Seldom/rarely -------------------------------------5  Never -----------------------------------------------6  Don’t know/don’t remember --------------------7 | | | |  | |
| d | With family members  *Please check ONLY ONE* | | Almost always/always ----------------------------1  Usually/most of the time -------------------------2  Often ------------------------------------------------3  Sometimes -----------------------------------------4  Seldom/rarely -------------------------------------5  Never -----------------------------------------------6  Don’t know/don’t remember --------------------7 | | | |  | |
| e | With others  *Please check ONLY ONE* | | Almost always/always ----------------------------1  Usually/most of the time -------------------------2  Often ------------------------------------------------3  Sometimes -----------------------------------------4  Seldom/rarely -------------------------------------5  Never -----------------------------------------------6  Don’t know/don’t remember --------------------7 | | | |  | |
| 220 | What type of discomfort do you typically feel immediately after taking shisha?  *(Multiple responses allowed)* | | Coughing ---------------------------------------------A  Choking -----------------------------------------------B  Light-headedness ---------------- -------------------C  Headache ---------------------------------------------D  Vomiting/nausea ------------------------------------E  Chest pain --------------------------------------------F  Bitter taste -------------------------------------------G  Loss of consciousness -----------------------------H  Frequent stooling ------------------------------------I  Others (Specify) ------------------------------------X  None --------------------------------------------------K  Don’t know ------------------------------------------Y  Refused -----------------------------------------------Z | | | |  | |
| 221 | Which substances have you ever used together with shisha?  *(OPTIONS NOT TO BE READ OUT. Enumerator should probe for more substances, one after another)*  *(Multiple responses allowed)* | | Cigarette   ---------------------------------------------A  Alcohol -----------------------------------------------B  “TomTom”/other type of “sweet” ----------------C  Beverages --------------------------------------------D  Brandy -----------------------------------------------E  Whisky -----------------------------------------------F  Red wine --------------------------------------------G  Other red wine --------------------------------------I  Gin ----------------------------------------------------J  Palm wine--------------------------------------------K  Cough syrup -----------------------------------------L  Codeine ----------------------------------------------M  Indian hemp/Marijuana/weed ---------------------N  Cocaine or heroin-----------------------------------O  Valium------------------------------------------------Q  Tramadol/Pentazocine------------------------------R  None --------------------------------------------------Y  Other (specify)---------------------------------------X | | | |  | |
|  | **CURRENT DAILY USERS AND CURRENTLY LESS THAN DAILY USERS ONLY** | | | | | |  | |
| 222 | When was the LAST time you used shisha, even one or two times? (Please choose the first answer that fits)  (Enumerator: Please choose the first answer that fits) | | Earlier today ----------------------------------------------1  Not today, but sometime during the past 7 days -----2  Not during the past 7 days, but sometime during the past 30 days-----------------------------------------------3  Not during the past 30 days, but sometime during the past 6 months----------------------------------------------4  Not during the past 6 months, but sometime during the past year------------------------------------------------5  1 to 4 years ago -------------------------------------------6  5 or more years ago --------------------------------------7  Don’t know ------------------------------------------------8  Refused ----------------------------------------------------9 | | | |  | |
| 223 | How frequent do you use shisha?  *(Enumerator to read options out)* | | At least once a month, but not daily ------------------1  At least once a week, but not daily --------------------2  At least once daily, or most days each week----------3 | | | |  | |
| 224 | On the days you use shisha, how frequent do you use shisha **per day**?  *(Enumerator to read options out)*  *(Only one response allowed)* | | About 1 time per day ------------------------------------1  About 2 times per day -----------------------------------2  About 3 times per day -----------------------------------3  More than 3 times per day ------------------------------4  None —----------------------------------------------------5  Don’t know ------------------------------------------------9 | | | |  | |
| 225 | On the days you use shisha, how long is your typical shisha use session? | | 0-10 minutes —----------------------------------------1  11-30 minutes —---------------------------------------2  31-60 minutes —---------------------------------------3  61-90 minutes —---------------------------------------4  121 or more minutes —-------------------------------5 | | | |  | |
| 226 | On the days you use shisha, how many number of bowls did typically you smoke per occasion? | | None —--------------------------------------------------1  One —---------------------------------------------------2  2-5 —----------------------------------------------------3  6-10 —---------------------------------------------------4  11-20 —-------------------------------------------------5  21-30 —-------------------------------------------------6  31 or more bowls —----------------------------------7 | | | |  | |
| 227 | On the days you use shisha, do you typically use shisha mainly when you are with people, mainly when you are alone, or do you smoke as often by yourself as with others? | | Mainly when with people -------------------------------1  Mainly when alone ---------------------------------------2  As often by yourself as with others --------------------3  Don’t know------------------------------------------------8  Refused ----------------------------------------------------9 | | | |  | |
| 228 | How frequent do you use flavoured shisha? | | Never -----------------------------------------------------1  Sometimes -----------------------------------------------2  Always ---------------------------------------------------3 | | | |  | |
| 229 | What is your favourite shisha flavour? | | Apple flavour--------------------------------------------1  Coconut flavour-----------------------------------------3  Strawberry flavour--------------------------------------4  Mint flavour --------------------------------------------5  Pineapple flavour --------------------------------------6  Vanilla flavour ------------------------------------------7  Chocolate flavour --------------------------------------8  Any flavour--------------------------------------------- 9  Other (Specify) ----------------------------------------96 | | | |  | |
| 230 | What time of the day is your use of shisha most frequent? | | Morning   -----------------------------------------------1  Afternoon -----------------------------------------------2  Evening -------------------------------------------------3  Refused -------------------------------------------------8 | | | |  | |
| 231 | Which of the following best describes the type of shisha you have used in the past 30 days?  *If you have used more than one type, please think about the one you use most often.* | | Ordinary shisha ------------------------------------------1  Shisha mixed with other substance -------------------2  Both ordinary and mixed shisha -----------------------3  Don’t know ----------------------------------------------8  Refused ---------------------------------------------------9 | | | |  | |
| 232 | What type of liquid was used in the “bowl” of the shisha that you used the last time? | | Water---------------------------------------------------1  Alcohol-------------------------------------------------2  Don’t know--------------------------------------------3  Others (Specify) --------------------------------------8 | | | |  | |
| 233 | Approximately how much do you spend on shisha per month in Naira? | | \|  \|  \|  \|  \|  \| \| --- \| --- \| --- \| --- \| --- \|   Naira | | | |  | |
| 234 | The last time you smoked shisha, how many other people did you share the same pipe  with, during the session? | | \|  \|  \| \| --- \| --- \| | | | |  | |
|  | **CESSATION OF SHISHA USE (CURRENT USERS ONLY - DAILY OR NOT DAILY)** | | | | | |  | |
|  | **The next questions ask about any attempts to stop using shisha that you might have made during the past 12 months**. | | | | | |  | |
| 235 | During the past 12 months, have you tried to stop using shisha | | Yes--------------------------------------------------------1  No---------------------------------------------------------2  Refused --------------------------------------------------9 | | | |  | |
| 236 | Which of the following influenced your decision to stop smoking shisha?  *(Tick as apply)*  *(Multiple responses allowed)* | | Personal Health concerns ------------------------------A  Costs ------------------------------------------------------B  Family pressure or disapproval -----------------------C  Smoking restrictions -----------------------------------D  Friends’ disapproval ------------------------------------E  Concern that your smoking is bad for others --------F  Wanting to set a good example for children? -------G  Others (Specify) ----------------------------------------H  Don’t know -----------------------------------------------I  Refused -------------------------------------------------J | | | |  | |
| 237 | Thinking about the last time you tried to quit, how long did you stop smoking? | | MONTHS ................................................................ 1  WEEKS ................................................................... 2  DAYS ...................................................................... 3  LESS THAN 1 DAY (24 HOURS) ….................... 4  DON’T KNOW ......................................................98  REFUSED ..............................................................99 | | | |  | |
| 238 | [ENTER NUMBER OF (MONTHS/WEEKS/DAYS)] | | \|  \|  \| \| --- \| --- \| | | | |  | |
| 239 | During the past 12 months, have you used any of the following to try to stop smoking tobacco? | | Counseling, including at a smoking cessation clinic--------------------------------------------------------------A  Nicotine replacement therapy, such as the patch or gum -------------------------------------------------------B  Other prescription medications? ----------------------C  Traditional medicines? ---------------------------------D  A quit line or a telephone support line? --------------E  Quit without assistance? -------------------------------F  Anything else? (Specify) ------------------------------X | | | |  |  |
|  |  |  |  |  |  |  |  |  |
| 240 | Which of the following best describes your thinking about quitting shisha use?  *I am planning to quit within the next month; I am thinking about quitting within the next 12 months; I will quit someday but not within the next 12 months; or I am not interested in quitting?* | | Quit within the next month.........…...…………….1  Thinking within the next 12 months .......................2  Quit someday, but not next 12 Months ………….3  Not interested in quitting ............……….………...4  Don’t know..............................................................9 | | | |  | |
| 241 | How easy or hard would it be for you to quit smoking shisha if you wanted to?  *Would you say very easy, somewhat easy, neither easy nor hard, somewhat hard, or very hard?* | | Very easy ............ ............ ................................... 1  Somewhat easy ........... ............ ......................... ..2  Neither easy nor hard .......... .......................….…3  Somewhat hard ................................................…4  Very hard .............................................................5  Don’t know ................. ..................................... ..9 | | | |  | |
|  | **ALL RESPONDENTS** | | | | | |  | |
| 242 | Have you smoked cigarettes before? | | Used in the past 30 days ---------------------------------1  Used but not in the past 30 days -----------------------2  Used but not in the past 12 months --------------------3  Never used ------------------------------------------------4  Refused ----------------------------------------------------9 | | | |  | |
|  | **(CURRENT SHISHA USERS ONLY )** | | | | | |  | |
| 243 | How often do you smoke shisha while smoking cigarettes? | | Never ------------------------------------------------------1  Rarely -----------------------------------------------------2  Sometimes ------------------------------------------------3  Always ----------------------------------------------------4 | | | |  | |
| 244 | When you are smoking cigarettes, what happens to your shisha smoking level?  (One response only) | | Decreases --------------------------------------------------1  Decreases slightly ----------------------------------------2  Stays the same --------------------------------------------3  Increases slightly -----------------------------------------4  Increases ---------------------------------------------------5  Don’t know------------------------------------------------8  Refused-----------------------------------------------------9 | | | |  | |
| 245 | On a scale of 0 to 10, with 0 being “not at all difficult” and 10 being extremely difficult, how difficult is it for you to use shisha without smoking a cigarette?  Enter number ……..number (0-10) | | \|  \|  \| \| --- \| --- \| | | | |  | |
|  | **ALL RESPONDENTS** | | | | | |  | |
| 246 | Have you drunk alcohol before? | | Used in the past 30 days ---------------------------------1  Used but not in the past 30 days -----------------------2  Used but not in the past 12 months --------------------3  Never used ------------------------------------------------4  Refused --------------------------------------------------99 | | | |  | |
|  | **CURRENT SHISHA USERS ONLY** | | | | | |  | |
| 247 | How often do you smoke shisha while drinking alcoholic beverages? | | Never ------------------------------------------------------1  Rarely -----------------------------------------------------2  Sometimes ------------------------------------------------3  Always ----------------------------------------------------4 | | | |  | |
| 248 | When you are drinking alcohol, what happens to your shisha smoking level?  (One response only) | | Decreases --------------------------------------------------1  Decreases slightly ----------------------------------------2  Stays the same --------------------------------------------3  Increases slightly -----------------------------------------4  Increases ---------------------------------------------------5  Don’t know------------------------------------------------8  Refused-----------------------------------------------------9 | | | |  | |
| 249 | On a scale of 0 to 10, with 0 being “not at all difficult” and 10 being extremely difficult, how difficult is it for you to use shisha without drinking alcohol?  Enter number …….. number (0-10) | | \|  \|  \| \| --- \| --- \| | | | |  | |
|  | **ALL RESPONDENTS** | | | | | |  | |
| 250 | How many of your closest friends have ever used shisha?  (Enumerator: enter 988 if don’t know) | \|  \|  \|  \| \| --- \| --- \| --- \| | | | | |  | |
| 251 | Does anyone in your family use shisha? | Yes -------------------------------------------------------1  No --------------------------------------------------------2  Not sure --------------------------------------------------3 | | | | |  | |
| 252 | How many people in your household use shisha?  *(Enumerator: enter 988 if don’t know)* | \|  \|  \|  \| \| --- \| --- \| --- \| | | | | |  | |
| 253 | Based on what you know, for each statement below, please indicate yes or no for each of the following statement:  (Enumerator is allowed to read out the statements one after the other) | Statement | | Yes | No | Don’t Know |  | |
|  |  | 1. If people are smoking cigarettes, I would rather be somewhere else | |  |  |  |  | |
|  |  | 1. If people are smoking shisha, I would rather be somewhere else | |  |  |  |  | |
|  |  | 1. If people are smoking cigarettes, it does not bother me | |  |  |  |  | |
|  |  | 1. If people are smoking shisha, it does not bother me | |  |  |  |  | |

# SECTION 3: REASONS FOR USE OF SHISHA *(CURRENT OR PAST USERS ONLY)*

| 301 | What are the reasons that you have used shisha?  *(Select one or more)* _____________________ | A friend used them --------------------------------------------A  A family member used them -----------------------------------B  To try to quit using other tobacco products, such as-cigarettes-------------------------------------------------------C  They cost less than other tobacco products, such as  Cigarettes- --------------------------------------------------------D  They are easier to get than other tobacco products, such as cigarettes----------------------------------------------------------E  I’ve seen people on TV, online, or in movies use them ---F  They are less harmful than other forms of tobacco, such as cigarettes----------------------------------------------------------G  They are available in flavours, such as mint, candy, fruit, or chocolate----------------------------------------------------------H  I can use them unnoticed at home or at school- ------------I  I can use them to do tricks---------------------------------------J  I was curious about them. -------------------------------------K  I enjoy the smell …………………………………………. L  I enjoy the taste …………………………………………. M  I used them for some other reason (specify): ---------------- L |
| --- | --- | --- |

# SECTION 4: ATTITUDE AND PERCEPTION TOWARDS SHISHA USE (CURRENT AND PAST USERS ONLY)

The following are some statements concerning shisha use. Please indicate whether you totally disagree, do not totally agree, more or less agree or fully agree or not.

1. **Attitudes Toward Smoking Scale (ATS-18) - Adapted**

| S/N |  | Totally disagree | Do not really agree | More or less agree | Agree | Fully agree |
| --- | --- | --- | --- | --- | --- | --- |
|  | **Adverse effects of smoking shisha** | | | | | |
| 401 | Shisha smoking is extremely dangerous to my health |  |  |  |  |  |
| 402 | Shisha smoking is ruining my health |  |  |  |  |  |
| 403 | My shisha smoke leaves an unpleasant smell |  |  |  |  |  |
| 404 | Shisha smoking gives me very bad breath |  |  |  |  |  |
| 405 | I spend too much money on shisha |  |  |  |  |  |
| 406 | My shisha smoke bothers other people a great deal |  |  |  |  |  |
| 407 | My shisha smoke is dangerous to those around me |  |  |  |  |  |
| 408 | Shisha smoking is bad for my skin |  |  |  |  |  |
| 409 | It bothers me to be dependent on shisha |  |  |  |  |  |
| 410 | I would have more energy if I did not smoke shisha |  |  |  |  |  |
|  | **Psychoactive benefits of smoking shisha** | | | | | |
| 411 | A shisha calms me down when I am stressed |  |  |  |  |  |
| 412 | Shisha calms me down when I am upset |  |  |  |  |  |
| 413 | A shisha helps me deal with difficult situations |  |  |  |  |  |
| 414 | After a shisha, I am able to concentrate better |  |  |  |  |  |
|  | **Pleasure of smoking shisha** | | | | | |
| 415 | I like the motions of smoking shisha |  |  |  |  |  |
| 416 | It feels so good to smoke shisha |  |  |  |  |  |
| 417 | I love smoking shisha |  |  |  |  |  |
| 418 | I like to hold a shisha (e.g vape or shisha pen etc) between my fingers |  |  |  |  |  |

1. **Social perceptions about shisha smoking**

| S/N |  | Totally disagree | Do not really agree | More or less agree | Agree | Fully agree |
| --- | --- | --- | --- | --- | --- | --- |
| 419 | Shisha is a sign of high social status |  |  |  |  |  |
| 420 | Shisha smoking is more socially acceptable compared to cigarette |  |  |  |  |  |
| 421 | Using shisha is a good opportunity to get together with family and friends |  |  |  |  |  |
| 422 | Shisha use helps people feel more comfortable at celebrations, parties, or in other social gatherings |  |  |  |  |  |
| 423 | Shisha’s smoke is more accepted by society than cigarettes’ smoke |  |  |  |  |  |
| 424 | Shisha smokers have more friends than those who do not smoke |  |  |  |  |  |
| 425 | Females are more comfortable in taking shisha compared to cigarette |  |  |  |  |  |
| 426 | People who matter most to me are pleased with me if I smoke shisha |  |  |  |  |  |
| 427 | My favourite famous figures or role model use shisha |  |  |  |  |  |
| 428 | Rich people use shisha more than poor |  |  |  |  |  |
| 429 | Shisha shows the person is adventurous |  |  |  |  |  |
| 430 | Shisha is cool and trendy |  |  |  |  |  |
| 431 | Stylish persons use shisha |  |  |  |  |  |
| 432 | Shisha is gaining popularity and many of my friends are using it |  |  |  |  |  |

# SECTION 5: CAPACITY TO DISCONTINUE SHISHA USE

**Adapting Smoking Self-efficacy questionnaire -SEQ-12:** The following are some situations in which certain people might be tempted to use shisha. Please indicate whether you are not sure at all, not very sure, more or less sure, fairly sure, absolutely sure that you could refrain from using shisha in each situation.

**(Applicable to Current Users Only)**

|  |  | Not at all sure | Not very sure | More or less sure | Fairly sure | Absolutely sure |
| --- | --- | --- | --- | --- | --- | --- |
| 501 | When I feel nervous |  |  |  |  |  |
| 502 | When I feel depressed |  |  |  |  |  |
| 503 | When I am angry |  |  |  |  |  |
| 504 | When I feel very anxious |  |  |  |  |  |
| 505 | When I want to think about a difficult problem |  |  |  |  |  |
| 506 | When I feel the urge to smoke |  |  |  |  |  |
| 507 | When having a drink with friends |  |  |  |  |  |
| 508 | When celebrating something |  |  |  |  |  |
| 509 | When drinking beer, wine, or other spirits |  |  |  |  |  |
| 510 | When I am with smokers |  |  |  |  |  |
| 511 | After a meal |  |  |  |  |  |
| 512 | When having coffee or tea |  |  |  |  |  |

# SECTION 6: KNOWLEDGE AND HEALTH PERCEPTIONS OF SHISHA USE

**(Applicable to current and past users only)**

| **NO.** | **QUESTIONS AND FILTERS** | **CODING CATEGORY** | **SKIP** |
| --- | --- | --- | --- |
| 601 | Based on what you know, does smoking shisha cause serious illness? | Yes------------------------------------------- 1  No-------------------------------------------- 2  Don’t Know--------------------------------98  Refused-------------------------------------99 |  |
| 602 | Based on what you know, does shisha contain a significant amount of tobacco? | Yes -------------------------------------------1  No --------------------------------------------2  Don’t Know ---------------------------------3 |  |
| 603 | Based on what you know, which one is more addictive between cigarettes and shisha? | Cigarettes much more addictive ----------1  Cigarettes slightly more addictive -------2  Shisha slightly more addictive ------------3  Shisha much more addictive --------------4  Both are equally addictive ----------------5 |  |
| 604 | Based on what you know or believe, does smoking shisha cause the following…? | Stroke (blood clots in the brain that may cause paralysis) ---------------------------- A  Heart attack ---------------------------------B  Lung cancer ---------------------------------C  Diabetes -------------------------------------D  Respiratory diseases -----------------------E  Sensitive towards light and sound ------- F  Ulcer -----------------------------------------G  Menstrual disorders ----------------------- H  Pregnancy hazards -------------------------I  Other? (Specify) ---------------------------X  None -----------------------------------------Z |  |
| 605 | Do you believe shisha is addictive? | Yes ------------------------------------------1  No -------------------------------------------2  Don’t Know -------------------------------98  Refused ------------------------------------99 |  |
| 608 | During the past 30 days, did you see any health warnings on shisha packages? | Yes------------------------------------------1  No-------------------------------------------2  Not sure------------------------------------8 |  |
| 610 | Do you think shisha smoking affects other non-smokers who are in the vicinity of those smoking shisha? | Yes -----------------------------------------1  No ------------------------------------------2  Don’t know -------------------------------8 |  |
| 612 | Compared to smoking cigarettes, do you think using shisha is less harmful, no different, or more harmful? | Less harmful than cigarette -----------1  No different -----------------------------2→  More harmful than cigarette ---------3 →  Don’t know -----------------------------8 → | 701  701  701 |
| 613 | Why do you think shisha smoking is less harmful than cigarette smoking?  (Enumerator can choose more than one  answer) | Shisha has an efficient filtration mechanism ---------------------------------A  The fruit flavour in shisha detoxifies the smoke ---------------------------------------B  The amount of cancerous substances is lesser in shisha smoke --------------------C  The amount of Nicotine is lesser in shisha smoke ------------------------------D  Chain smokers consume more than 20 cigarettes per day but even habitual Shisha users practise it not more than once daily ----------------------------------E  Shisha smoking is less irritating and thus less toxic to the respiratory tract --------F |  |

# Section 7: STRESS, ANXIETY AND DEPRESSION

**(All Respondents)**

**Perceived Stress Scale**

The next set of questions asks you about your feelings and thoughts during last month. In each case, you will be asked to indicate how often you felt or thought in a certain way (i.e. would you say never, almost never, sometimes, fairly often, very often?).

| **S/N** | **Questions** | **Never** | **Almost never** | **Sometimes** | **Fairly often** | **Very often** |
| --- | --- | --- | --- | --- | --- | --- |
| 701 | In the last month, how often have you felt that you were unable to control the important things in your life? |  |  |  |  |  |
| 702 | In the last month, how often have you felt confident about your ability to handle your personal problems? |  |  |  |  |  |
| 703 | In the last month, how often have you felt that things were going your way? |  |  |  |  |  |
| 704 | In the last month, how often have you felt difficulties were piling up so high that you could not overcome them? |  |  |  |  |  |

**Patient Health Questionnaire-2 (PHQ-2):** Over **the last 2 weeks**, how often have you been bothered by any of the following problems?

Would you say you were not bothered at all, bothered for several days, more than half days, or nearly every day?

| **S/N** | **Questions** | **Not at all** | **Several days** | **More than half the days** | **Nearly every day** |
| --- | --- | --- | --- | --- | --- |
| 705 | Little interest or pleasure in doing things |  |  |  |  |
| 706 | Feeling down, depressed, or hopeless |  |  |  |  |

**General Anxiety Disorders-2 (GAD-2):** Over **the last 2 weeks**, how often have you been bothered by any of the following problems? Would you say you were not bothered at all, bothered for several days, more than half days, or nearly every day?

|  |  | **Not at all** | **Several days** | **More than half the days** | **Nearly every day** |
| --- | --- | --- | --- | --- | --- |
| 707 | Feeling nervous, anxious or on edge |  |  |  |  |
| 708 | Not being able to stop or control worrying |  |  |  |  |

# SECTION 8: MEDIA AND KNOWLEDGE OF REGULATION OF SHISHA USE IN NIGERIA

**(Applicable to current and past users only)**

| **NO.** | **QUESTIONS AND FILTERS** | **CODING CATEGORY** | **SKIP** |
| --- | --- | --- | --- |
| 801 | Are you aware of any law or regulation “generally” guiding the use of tobacco in your community, state, or Nigeria in general? | Yes -----------------------------------------1  No ------------------------------------------2 |  |
| 802 | Are you aware of any law or regulation “specifically” guiding the use of shisha in your community, state, or Nigeria in general? | Yes -----------------------------------------1  No ------------------------------------------2→ | 804 |
| 803 | To what extent do you think the general public are complying with this law or regulation on shisha use in your area? | Very high ----------------------------------1  High ----------------------------------------2  Moderate -----------------------------------3  Low -----------------------------------------4  Very low -----------------------------------5 |  |
| 804 | Would you favour or oppose a law prohibiting all advertisements for shisha products? | Favour----------------------------------------1  Oppose---------------------------------------2  Don’t Know --------------------------------98  Refused -------------------------------------99 |  |
| 805 | Would you support or oppose a law that would prohibit smoking shisha in all indoor workplaces and public places such as restaurants/clubs/bars? | Support -----------------------------------1  Oppose------------------------------------2  Don’t Know------------------------------8 |  |
| 806 | In the last 30 days, have you noticed any advertisements or signs promoting the shisha in the following places?  **Options for each response category:**  Yes -----------------------------------------1  No ------------------------------------------2  Not Applicable----------------------------7  Refused ------------------------------------9 | a. In stores where shisha are sold  b. Television  c. Radio  d. Billboards  e. Posters  f. Newspaper or magazine  g. Cinema  h. Internet  i. public transportation vehicles or stations  j. Public walls  Z. Anywhere else (Specify) |  |
| 807 | In the last 30 days, did you notice any health warnings on packages of shisha tobacco or charcoal, or the waterpipe instrument? | Yes-----------------------------------------1  No------------------------------------------2  Did not see any shisha packages ------3 |  |
| 808 | In the last 30 days, have health warnings on shisha packages led you to think about quitting? | Yes-----------------------------------------1  No------------------------------------------2  Don’t know-------------------------------98  Refused -----------------------------------99 |  |
| 809 | In the last 30 days, have you noticed any information about the dangers of smoking shisha or that encourages quitting in any of the following places? media outlets such Newspaper, radio, Television, Billboard, etc.  (Multiple responses allowed) | Newspaper ----------------------------------A  R Television --------------------------------B  Radio ----------------------------------------C  Billboards -----------------------------------D  Somewhere else (Specify) ----------------E |  |

#

# SECTION 9: AIRTIME NUMBER AND CONTACT INFORMATION

**(Applicable to all respondents)**

| 901 | Just to confirm, what’s the best number to reach you if we have follow-up questions directly? | \|____\|____\|____\|____\|____\|____\|____\|____\|____\|____\| |  |
| --- | --- | --- | --- |
| 902 | Could you give me another phone number where we could reach you in case we cannot connect with you at your primary number? | Yes -------------------------------------------------------1  No --------------------------------------------------------2→ | 904 |
| 903 | Enter phone number 2 here (secondary number) | \|____\|____\|____\|____\|____\|____\|____\|____\|____\|____\| |  |
| 904 | We have come to the end of the interview. As mentioned earlier, we have a token of appreciation in form of airtime which will be sent directly to your phone  What is the phone number that you would like this airtime to be sent to? | \|____\|____\|____\|____\|____\|____\|____\|____\|____\|____\| |  |

# APPENDIX A: SHISHA SURVEY AND THEORY OF PLANNED BEHAVIOUR (TPB) MAPPING

|  | **What** | **How** | **Who** | **Supported by qualitative study?** |
| --- | --- | --- | --- | --- |
| **Demographics and socio-economics** | | | | |
|  | Age of respondents at last birthday | GATS |  | Yes |
|  | Gender | GATS |  | Yes |
|  | Urban/Rural residence | NBS |  | Yes |
|  | Highest level of education of respondent | GATS |  | Yes |
|  | Marital status | GATS |  | No |
|  | Main work status over past 12 months | GATS |  | No |
|  | Wealth status (household ownership of items) | GATS |  | No |
|  | Ethnic background of respondents | NDHS |  | No |
|  | Religion | GATS |  | Yes |
| **Shisha use behaviour and patterns** | | | | |
|  | Shisha smoking (ever use) | CDC-NYTS | Both smokers and non-smokers | No |
|  | Shisha smoking (current smoking status) | GATS | Ever users (Current and stopped) | Yes |
|  | Reasons for quitting shisha use; last time used | GATS | Past users | Yes |
|  | Shisha use initiation (age at initiation; years of initiation, where, with whom) | GATS | Current and past users | Yes |
|  | Shisha use initiation (where first heard, about shisha, emotional state prior to use) | Specific question from Qualitative findings |  | Yes |
|  | Shisha use initiation (Any discomfort experienced after first use) | Wong et.al 2017; and specific question from Qualitative findings and |  | Yes |
|  | Company at initiation | Holtzman et al. |  | Yes |
|  | Shisha use (last time used, frequency; duration, number used per occasion, with whom, years used shisha) | QUESTIONS FROM GATS, CDC (NYTS), Holtzman et al | Current users | Yes |
|  | Type of discomfort experienced after use | Pradnya & Shruti | Current and past users | Yes |
|  | Flavoured shisha (usage & frequency) | GATS and Holtzman et al |  | Yes |
|  | Favourite shisha flavour | Specific question from Qualitative findings |  |  |
|  | Where (where used, where preferred to use shisha) | GATS |  | Yes |
|  | When (time of day; day of the week) | Specific question from Qualitative findings | Current Users | Yes |
|  | With whom (social smoking) | Moran et al; Hamadeh et al | Current and past users | Yes |
|  | Type used, how much, co-consumption with other substance | Specific question from Qualitative findings |  | Yes |
|  | Usual place of purchase and smoking | Hamadeh et al |  | No |
|  | Reasons for using shisha | CDC NYTS and Specific question from Qualitative findings |  | Yes |
| **Individual's Attitudes (the sum of all our knowledge, attitudes, prejudices , mood …. positive and negative that we think of when we consider shisha user)** | | | | |
|  | Attitudes towards shisha smoking | Adapt ATS-18 | Current Smokers | Yes |
|  | Shisha Knowledge and health perception | Questions from GATS, Jawaid et al, Holtzman et al, Wong et al,Algahtani | Both smokers and non-smokers | Yes |
| **Subjective norms (Individual’s views on how close friends, family or the society perceive shisha use - significant others' judgement** | | | | |
| Social smoking | Acceptability, benefits and comfortability offered by shisha usage (questions 419-426) | Questions from GATS; Anjum Q et al.; Algahtani; Specific question from Qualitative findings | Current and past shisha Users | Yes |
| Social support | Significant others are pleased if smoke shisha | Maibach et al., 1996 | Current and past shisha Users | To some extent |
| **Descriptive norms (Individual’s views about use of shisha by most people or important figures in the society)** | | | | |
| Important figures’ behaviour | Used by role models; used by the rich, adventurous and stylish people etc. (Questions 427-432) | Questions from Wong et al.; Algahtani | Current and past shisha Users | To some extent |
| Friends and family behaviour | Number of smokers in the household; Number of close friends who smoke shisha; | Specific question from Qualitative findings | All respondents | To some extent |
| **Perceived behavioural control (Perceived ability of shisha users to refuse or discontinue smoking shisha)** | | | | |
|  | Cessation of shisha use (Quit attempt, methods used during quit attempt, quite intentions, reasons, duration of cessation, and perceived difficulty in quitting) | GATS |  | To some extent |
| **Self-efficacy (Perceived capability to overcome certain situations or conditions that may act as a barrier to discontinuation of shisha use)** | | | | |
|  | Self efficacy | Self-efficacy questionnaire-12 | Current smokers | To some extent |
|  | Regulation | GATS | Both smokers and non-smokers | To some extent |
| **Others** | | | | |
|  | Alcohol and Cigarettes use | Moran et al | Both smokers and non-smokers | Yes |
|  | Co-consumption with alcohol and Cigarettes shisha | Noreen et al | Current Smokers | Yes |
|  | Stress | PSS-4 | Both smokers and non-smokers | Yes |
|  | Depression | PHQ-2 | Both smokers and non-smokers | Yes |
|  | Anxiety | GAD-2 | Both smokers and non-smokers | Yes |
