# Supplementary material for: Reasons for shisha smoking: Findings from a mixed methods study among adult shisha smokers in Nigeria
Source: PLOS Glob Public Health. 2024 Feb 2;4(2):e0002853. doi: 10.1371/journal.pgph.0002853 (PMC10836660; doi:10.1371/journal.pgph.0002853)
Supplement: S2 Text — (DOCX) [file pgph.0002853.s004.docx]

**Reasons for shisha smoking: findings from a mixed methods study among adult shisha smokers in Nigeria**

**Interview Guide: In-depth Interviews**

**State:** _______________ **Date of interview:** ____________________

**Respondent’s Background Information:**

| Age | Gender (Male/Female) | Ethnicity | Location (town/community name) | Type of residence (Rural/Urban) | Highest Level of Education | Religious Affiliation |
| --- | --- | --- | --- | --- | --- | --- |
|  |  |  |  |  |  |  |

**Questions**

**Icebreaker**

1. Please can you tell me about yourself? *(no name please, just your likes, favourite food, hobbies etc.)*

**Respondent’s background and history of shisha smoking**

1. Please can you tell me how you started using shisha?
   1. Probe for the social circumstances e.g. when, where, among whom, what occasion etc and who introduced you to shisha (what is the relationship of this person to you?).
   2. Probe for Parents’ or carers’ shisha use (or tobacco use), individual smoking history (initiation/increase/decrease/quit, etc), daily and other context of smoking,
   3. Probe for use by significant others (e.g., use among family members (siblings, parents) or close friends, roommates, particularly cohabiting),
   4. How often do you smoke shisha (weekly/ monthly estimates)? for how long on the average per session? Are there any emotional circumstances that influence when you smoke shisha?
   5. Probe for whether ‘mood’ (e.g., feelings of depression, anxiety, happiness, stress) has any impact on whether they smoke Shisha or how much they smoke?
   6. Probe for smoking shisha with others, like friends, family members, significant others, mentors?
   7. Probe for the type of shisha often smoked by the respondent. (sweetened, flavoured, mixed with other substances, brands, if known).
   8. Probe for knowledge of other types and preferences. Probe for reasons for preference, content, influences from the media, mentors, friends, significant others or availability.
2. Please share with me what your experience was like during your first attempt smoking shisha?
   1. Probe for the immediate feelings, was it enjoyable or distressing
   2. What made you continue smoking it?
3. Please tell me where you often smoke shisha?
   1. Probe for home, office, among friends, hotels, restaurants, bars, clubs etc.
   2. Probe for preferred places and reasons for such preferences. If outside home, probe for how often he/she goes out and visit the preferred places mentioned?
   3. Probe for at what time of the day; and on which day(s) he/she usually goes there? Probe for living arrangements (if respondent lives alone or among family members/friends)
   4. Probe for typical expenditure per week or month (estimates) on shisha, location of purchases, and awareness of shisha advertising

**Perceived Benefits from smoking shisha**

1. Please share with us the benefits you enjoy from smoking shisha.
2. Are there any other benefits from smoking shisha? Where did you learn this from? Friends, relatives or other places (media, online media, print media or electronic media)

**Awareness of health implications of smoking shisha**

1. Please can you share with us any health concerns you currently have? Probe if currently suffering from symptoms of breathlessness/light-headedness, chest pain, recurrent headaches, high blood pressure, diabetes, recurrent episodes of depression or anxiety, menstrual disorders (if female), any other chronic illnesses like HIV, Cancer, or kidney diseases? Probe on future perceived health status
2. Please can you share with us any health implications of smoking shisha? Probe for both perceived positive and negative implications. Probe for sources of knowledge and why do you persist despite the awareness of some of the negative implications (if negative health implications are mentioned)?
3. Please tell me what is the impression of your friends and/ relatives about your smoking shisha? Probe for specific concerns raised by these significant others (finance, health, etc) and how have the concerns affected your smoking shisha?
4. Please tell me if you think shisha smoking affects other non-smokers who are in the vicinity of those smoking shisha?
   1. Probe perceptions of how this can affect non-smokers, either their health/ perception of shisha smoking/other social or psychological effects.
5. What do the public, especially non-shisha smokers say about those who smoke shisha? Probe for any positive and negative attitude towards shisha smokers

**Perceptions towards selected health risks of Shisha smoking**

1. I want to ask you about your opinion on the following statements. For each of the statement, kindly indicate whether you:
   1. Strongly agree
   2. Agree
   3. Neither agree nor disagree
   4. Disagree
   5. Strongly disagree

**Facilitator:** Read out the following questions one after the other.

**Note taker:** Record response in front of each question.

- 1. Shisha smoking has no negative health effects
  2. Shisha smoking improves my body functioning
  3. Shisha smoking is healthier than cigarette smoking
  4. Shisha smoke can affect the health of non-smokers in the vicinity of shisha smoking
  5. Smoking Shisha by pregnant women does not harm their babies

**Use of sundry substances and risky behaviours**

1. Please tell me about your cigarette smoking habit. When did you start smoking cigarettes?
   1. If currently smoking Shisha, probe for frequency and quantity per day or per week.
   2. If stopped, probe for reasons for discontinuing smoking cigarette, and
   3. if never smoked cigarette, probe for reasons for non-smoking
2. Please share with me your experiences at using other substances such as Indian hemp or marijuana, cocaine or heroin, prescription medicines like codeine, “cough syrup” valium, tramadol or pentazocine. Which ones do you use?
   1. If using any of the other substances, probe for duration of usage for each of them If more than one) How often do you use them?
   2. Do you use them at the same time as the shisha/together?
   3. For those who use at the same time or together, probe whether their shisha use changes in any way (e.g., increase or decrease in the amount smoked) if co-consuming, compared to when they are just having shisha on its own.
3. Please share with me your drinking habits (probe for kind of alcohol respondent consumes, brands, E.g. Local produce like local gin/palm wine, Brandy, Whisky, Wine (red/white), other types? What quantity do you consume daily or weekly? Probe:
   1. How long have you been drinking these beverages?
   2. Do you drink these beverages at the same time as smoking shisha? Can you share your experiences with me?
   3. For those who sometimes use shisha when they are drinking alcohol, probe whether their shisha use changes in any way (e.g., increase or decrease in the amount smoked), if co-consuming with alcohol, compared to when they are just having shisha on its own.
4. Please share with me any other activities you engage in while smoking shisha?

**Regulations on shisha use in Nigeria**

1. Please share with me any guidelines on use of tobacco products (shisha, cigarettes) in your community, state, or Nigeria in general. If any, probe for the extent to which retailers of tobacco products (i.e. social or night clubs, bar, pub, cafes, and restaurants) are following the guidelines on tobacco products like shisha in their community/state?

**Questions/Comments:**

1. Do you have any questions or additional comments to increase our understanding of the use of shisha in your community/area/state?
